# Brain-like border ownership signals support prediction of natural videos

**DOI:** 10.1016/j.isci.2025.112199

**Published:** 2025-03-11

**Authors:** Zeyuan Ye, Ralf Wessel, Tom P. Franken

**Affiliations:** 1 Department of Physics, Washington University in St. Louis, St. Louis, MO 63130, USA; 2 Department of Neuroscience, Washington University in St. Louis, St. Louis, MO 63110, USA

**Keywords:** Neuroscience, Behavioral neuroscience, Social sciences

## Abstract

To make sense of visual scenes, the brain must segment foreground from background. This is thought to be facilitated by neurons that signal border ownership (BOS), which indicate which side of a border in their receptive field is owned by an object. How these signals emerge without a teaching signal of what is foreground remains unclear. Here we find that many units in PredNet, a self-supervised deep neural network trained to predict future frames in natural videos, are selective for BOS. They share key properties with BOS neurons in the brain, including robustness to object transformations and hysteresis. Ablation revealed that BOS units contribute more to prediction than other units for videos with moving objects. Our findings suggest that BOS neurons might emerge due to an evolutionary or developmental pressure to predict future input in natural, complex dynamic environments, even without an explicit requirement to segment foreground from background.

## Highlights

• Neural network trained for video prediction develops border ownership (BOS) units
• The network’s BOS units behave similarly to BOS neurons in the primate brain
• Ablating these BOS units affects prediction more than ablating non-BOS units

## Teaser

Neuroscience; Sensory neuroscience; Cognitive neuroscience

## Introduction

**Figure 1:**
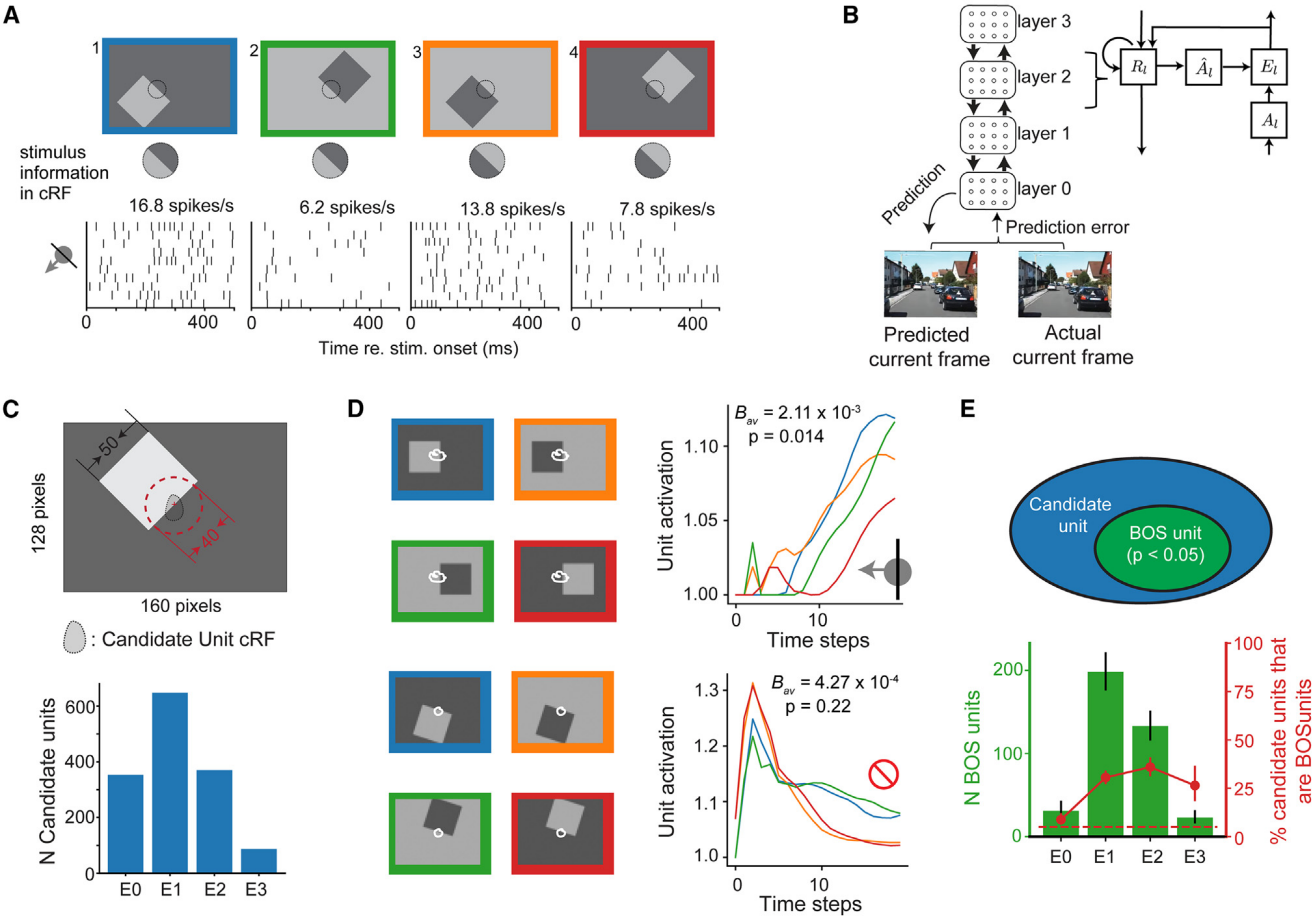
Border ownership (BOS) signals emerge in PredNet (A) An example unit in the primate visual cortex that is selective for BOS. The unit has different responses depending on the BOS, even though the image pixels in its classical receptive field (cRF) are identical for panel 1 and 2, and for panel 3 and 4. The preferred side of ownership is the same for borders in the cRF with a different contrast polarity (the unit fires more to scene 1 than to scene 2, and more to scene 3 than to scene 4). Arrow on the bottom left indicates the side of BOS that this unit prefers. Figure adapted from Franken and Reynolds (2021).^3^ (B) PredNet is an artificial neural network designed for video prediction. At each time step, the model operates by updating unit activities sequentially from the top layer (layer 3) to the bottom layer (layer 0), generating a prediction of the current video frame. The prediction error is then fed forward to layer 3. Each layer contains four modules (${\hat{A}}_{l},A_{l},E_{l}$, and $R_{l}$ where l=0,1,2,3 indicates layer index, see STAR Methods) (C) Candidate units in PredNet are defined as units whose cRF overlaps with the central border but not with any of the square’s other borders (see STAR Methods). Bottom: the number of candidate units in *E* modules across different layers. See Figure S2 for *R* module data. (D) Responses of two example units (module *E*_*2*_), with white contours indicating the cRF. $B_{av}$ measures, for each unit, the selectivity for BOS across different square orientations (see STAR Methods). Colored lines indicate the response to the different stimulus conditions (colors indicate for each response function to which of the stimulus panels on the left it corresponds) for one orientation. p value (two-tailed) was computed by comparing $B_{av}$ to that obtained by shuffling the BOS labels (permutation test, see STAR Methods). BOS units are defined as those units for which this value is smaller than 0.05. (E) Number (green bars) and percentage of BOS units (red data points) in E-module in different layers. Error bars indicate 95% confidence intervals. Horizontal dashed line indicates the chance level for the percentage of BOS units (5%). See also Figures S1–S8.

To understand the world around us, we parse incoming visual information into an organized collection of objects. In primate animals, this capability is thought to be facilitated by neurons in the early areas in visual cortex that encode border ownership (BOS).^1^^,^^2^^,^^3^^,^^4^ These neurons fire more to an identical border in their classical receptive field (cRF) depending on which side owns the border, even though the contextual information that defines the side of foreground occurs far outside of the cRF (Figure 1A). This selectivity extends to natural images^5^^,^^6^ and the preferred side of ownership corresponds to the side that is near when varying depth.^7^ Psychophysics and imaging studies support that BOS neurons also exist in the human brain.^8^^,^^9^^,^^10^^,^^11^ It is unknown under which conditions BOS signals emerge in neural networks. Artificial neural networks (ANNs), including deep neural networks, are great tools to study such “why” questions of how the brain works, because they enable to test whether a particular neural phenomenon results from optimization for a specific task.^12^

It seems intuitive to hypothesize that BOS signals emerge in ANNs when they are explicitly trained on scene segmentation, given that this is assumed to be the primary role of such signals in the brain.^13^ A recent study indeed found that units selective for BOS occur in a supervised ANN trained to segment handwritten digits (a processed MNIST dataset^14^) from background.^15^ Another study found BOS signals in a feedforward ANN trained to perform scene segmentation, although these signals differed from those in the brain in several ways, including the lack of size invariance.^16^ Moreover, such supervised learning has been criticized as biologically highly implausible because it requires a large number of explicitly segmented labels that is unrealistic in brain development.^17^^,^^18^ Another study found that BOS signals can arise in an unsupervised ANN trained to develop translation invariance for an object presented in isolation, but this mechanism failed for scenes with more than one object,^19^ as opposed to BOS signals in the brain.^20^ Furthermore, these ANNs can often only process simple artificial datasets, unlike neural networks in modern deep-learning frameworks or the human brain, which are high performing on realistic natural visual inputs.^21^^,^^22^^,^^23^ It thus remains poorly understood when BOS signals emerge in neural networks.

Prior neurophysiological studies provide hints that BOS signals in the brain might be especially useful to process dynamic visual input. BOS signals persist when the defining contextual information disappears (thus even though the border has become ambiguous in terms of ownership).^24^ Moreover, these persistent BOS signals can transfer to other neurons with eye movements (even though those neurons never signaled the side-of-ownership while the contextual information was still present).^25^ These phenomena suggest that BOS neurons help provide perceptual continuity in dynamic conditions. This motivated us to study whether BOS signals emerge in an ANN trained to predict future visual input for natural videos. We studied PredNet, a deep neural network with an architecture inspired by predictive coding.^18^^,^^26^^,^^27^^,^^28^^,^^29^ PredNet was trained on a dataset of natural videos captured by car-mounted cameras (KITTI^30^) to predict the next video frame. Our *in-silico* experiments demonstrate that a significant fraction of units in PredNet exhibit BOS signals. Moreover, these BOS units share several properties with BOS neurons in the brain. Finally, ablating PredNet’s BOS units increased prediction error more than ablating the same number of non-BOS units. These findings indicate that BOS units with brain-like properties can emerge to assist in prediction even if there is no explicit need to segment foreground from background. The need to predict future input in natural videos is thus a potential mechanism that could explain how BOS neurons emerge in a self-supervised way. These scene segmentation signals in the brain, typically considered an example of a ventral “what” stream operation, might thus be more involved in processing dynamic aspects of visual input than is typically assumed.

## Results

### BOS signals emerge in an artificial network trained to predict the next frame of natural videos

To study the role of BOS signals in the processing of complex dynamic input we employed PredNet, a hierarchical ANN introduced by Lotter et al.^26^ (Figure 1B). PredNet comprises four layers, with four modules per layer: the representation ($R_{l}$), the predicted output (${\hat{A}}_{l}$), the prediction target ($A_{l}$), and the prediction error ($E_{l}$) modules. At each time step (see STAR Methods), signals propagate from the top layer to the zeroth layer, resulting in a prediction for the next video frame in ${\hat{A}}_{0}$. This prediction is then compared to the actual next frame provided in $A_{0}$. The prediction error signal subsequently propagates from the zeroth layer to the top layer. The network was trained to minimize the prediction error of videos in the KITTI dataset,^30^ which were captured by car-mounted cameras in various urban and rural settings in Germany.

We tested whether BOS units exist in PredNet by doing an *in-silico* experiment that is analogous to the neurophysiological studies on BOS^1^^,^^3^^,^^31^^,^^32^ (Figure 1A). We measured the cRF of each unit using sparse noise stimuli (Figure S1). First we identified candidate units for BOS tuning. For a unit to be a candidate unit, the cRF needed to include the center of the square’s central border (i.e., the border positioned at the scene center) but exclude any other border of the square (Figure 1C; STAR Methods). This criterion is similar to that used in neurophysiological studies on BOS.^1^^,^^3^ We found tens to hundreds of candidate units in different PredNet modules (Figure 1C; Figure S2A). We then analyzed the response from candidate units to the standard full square scenes. Each standard full square scene was presented to PredNet as a sequence of 20 identical images (20 time steps). Predictions of PredNet to example stimuli can be found in Figure S3. Figure 1D shows responses from two example candidate units. The top unit exhibited a larger response when the square was positioned on the left side of the central vertical border compared to the right side, irrespective of the contrast polarity across the border (i.e., blue vs. green and orange vs. red). This unit thus prefers that the border in its cRF is owned by a square on one side, similar to BOS neurons in the primate visual cortex.^1^^,^^3^ In contrast, the bottom unit did not exhibit a clear difference: its response was very similar for stimuli with opposite border ownership but identical contrast polarity of the central border. To quantify BOS tuning for each unit, we first computed the unit’s difference in response between stimuli of opposite border ownership across different contrast polarities, and divided it by the sum of the responses, resulting in the BOS index (BOI). The BOS index was then averaged across all square orientations, resulting in $B_{av}$ (see STAR Methods). The statistical significance of $B_{av}$ was determined by comparing it to a null distribution obtained by shuffling the stimulus labels. A candidate unit with a *p* value smaller than 0.05 was defined as a BOS unit; otherwise, it was defined as a non-BOS unit. The top unit in Figure 1D has a statistically significant $B_{av}$ and is therefore a BOS unit, while the bottom unit is a non-BOS unit. The BOS unit showed a rising temporal response over time, whereas the non-BOS unit showed a response that quickly decayed after the early peak, but this was not a systematic difference in the population of units (Figure S4).

We conducted a population analysis of $B_{av}$ across all candidate units ($B_{av}$ values shown in Figures S2 and S5). We find that 20%–40% of candidate units in *E*_*1*_, *E*_*2*_, and *E*_*3*_ have significant $B_{av}$ values, and we define these as BOS units (red dots in Figure 1E). This is larger than in module $E_{0}$ (8.7%, 95% confidence interval [6.2%, 12%]). The percentage of BOS units among candidate units was also substantially higher than expected by chance in modules *R*_*1*_ and *R*_*2*_, but not in *R*_*0*_ (Figure S2; note that the absolute number of candidate units was much larger in E modules than in R modules, compare Figure 1C and Figure S2A; this is because R module units have larger cRF sizes [Figure S6]; *R*_*3*_ had only a small number of candidate units). The distribution of BOS units in PredNet’s hierarchy is reminiscent of the distribution of BOS neurons in the primate visual cortex, which are less prevalent in areas closer to the sensory input (V1) than in downstream areas (V2 and V4).^1^^,^^3^^,^^4^

We wondered whether the emergence of BOS units is critically dependent on the frame rate of the training videos (10 frames per second). We therefore trained an untrained instance of PredNet, but downsampled the videos such that the frame rate is 5 frames per second (200 ms per frame). We find a substantial and statistically significant percentage of BOS units in this network (Figure S7). The emergence of BOS-units in PredNet is thus robust to changing the video frame rate.

Together these findings suggest that the need to predict future input in dynamic videos drives the emergence of BOS units in PredNet. To test this idea, we trained an untrained instance of PredNet to reproduce images rather than to predict future dynamic input, by using sequences that consist of identical frames (see STAR Methods). This network does not contain a significant percentage of BOS units in either the E or the R modules (Figure S8), supporting the importance of video prediction.

### PredNet’s BOS signals are robust to scene variations common in natural object transformations

**Figure 2:**
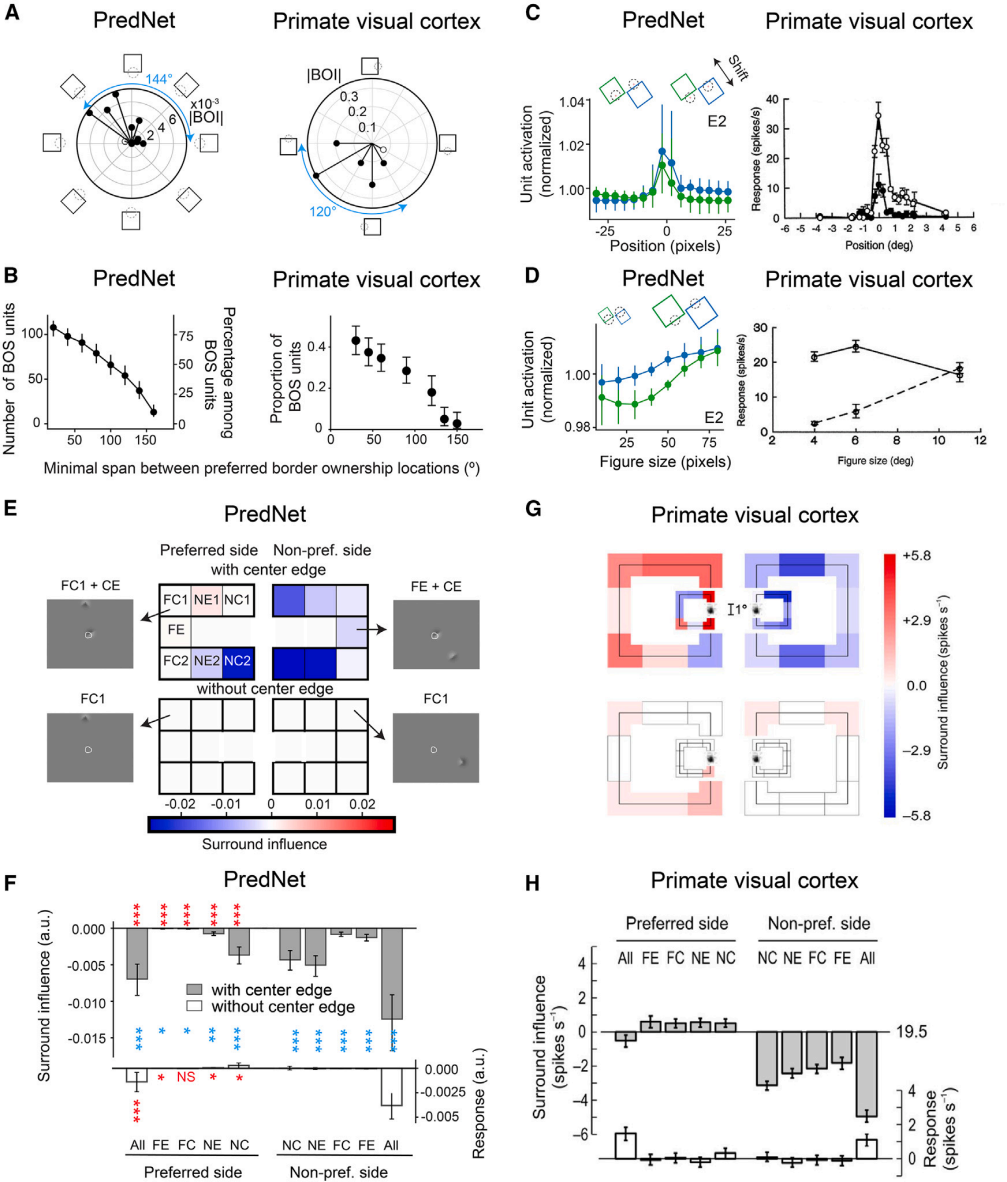
BOS units in PredNet share several properties with BOS neurons in the brain (A) Border ownership index (BOI) at different orientations. Vector magnitude represents the absolute value of BOI, i.e., the difference in unit response to scenes with squares that share a border with a given orientation, but for which the square is positioned on opposite sides of that border (thus a pair of stimulus cartoons on opposite sides of the polar plot), divided by their sum (see STAR Methods). Vector angle is such that the vector points toward the stimulus cartoon with the preferred side of BOS for that border orientation. Left: example of a BOS unit in PredNet. Right: BOS neuron recorded in macaque area V4 (adapted from Franken and Reynolds [2021]^3^). Filled symbols in both panels indicate for which orientations the BOI was significantly different from 0 (permutation test *p* < 0.05). Blue text indicates the BOS span of a unit, which is the angle between the preferred object locations for orientations with statistically significant BOI (STAR Methods). (B) The y axis illustrates the number/percentage of BOS units whose spans equal or exceed the span indicated by the x values. Error bars indicate 95% confidence intervals. Left: BOS units in module $E_{2}$ in PredNet (*n* = 133 BOS units; see Figure S9A for other modules). Right: population data from BOS neurons in macaque area V4 (adapted from Franken and Reynolds [2021]^3^). (C) Left: for each BOS unit, squares were generated with different positions as indicated in the cartoon. The blue and green traces represent normalized population responses (see STAR Methods) to opposite BOS (blue corresponds to the preferred BOS derived from responses to the standard square set). Dots and error bars show the median, first, and third quantiles across the population of BOS units (*n* = 133 BOS units in module E2). The average cRF diameter is 22.54 pixels. Right: responses from a BOS neuron in macaque V2 for different square positions. The two traces indicate opposite BOS. Dots and error bars represent mean firing rates and SEMs across trials (reprinted from Zhou et al. [2000].^1^ Copyright 2000 Society for Neuroscience). (D) Identical to (C) but square size was varied instead of square position (*n* = 133 BOS units in module E2). Right panel reprinted from Zhou et al. (2000).^1^ Copyright 2000 Society for Neuroscience. (E) Response of an example BOS unit in PredNet (module $E_{2}$) to square fragments. Top half shows responses to a square fragment in the surround paired with the border in the cRF. Bottom half shows responses to square fragments in the surround without the border in the cRF. Gray panels show example scenes (white outline: cRF). Colors of the central panels indicate the surround influence. The surround influence is the unit’s response to a scene with a square fragment in the surround at the position indicated by the letter codes (also symbolized by the panel’s position), subtracted by that to a scene without the square fragment. Letter codes: NC, near corner; NE, near edge; FC, far corner; FE, far edge. Numbers indicate different positions of the fragment, e.g., NC1 and NC2 refer to each of the near corners on opposite ends of the central border. (F) Means and 95% confidence intervals (i.e., 1.96 times SEM) of surround influence across all BOS units in module $E_{2}$ (*n* = 71; see Figure S10 for other modules). NC is the average of NC1 and NC2, and the same was done for NE and FC. “All” represents the surround influence when all square segments were shown (top), or all square segments except the center edge were shown (bottom). Red text indicates whether the surround influence for a particular condition is significantly larger on the preferred side than on the non-preferred side. For without-CE scenes the response is quantified as the difference in activity between the without-CE scene and a full gray scene. Blue text indicates whether the absolute value of surround influence of with-CE scenes is significantly larger than without-CE scenes. Wilcoxon signed-rank test. ∗∗∗: *p* < 0.001; ∗∗: *p* < 0.01; ∗: *p* < 0.05; NS: no significance. Outlier units (see STAR Methods) were removed to compute mean and SEM but included in the statistical tests. (G) Similar to (E) for a BOS neuron in the macaque visual cortex (reprinted from Zhang et al. [2010]^33^). Two different square sizes were evaluated, for which surround influence is plotted separately as the smaller and larger panels. (H) Similar to (F), for BOS neurons in the macaque visual cortex (reprinted from Zhang et al. [2010]^33^). (Data show linear estimates and 95% confidence intervals). See also Figures S9 and S10.

We explored the robustness of these BOS signals to the same scene variations that have been used in neurophysiological studies on BOS neurons: square orientation, position, and size.^1^^,^^3^ Figure 2A (left) shows the BOI for different square orientations for an example BOS unit in PredNet. Vector length indicates the absolute value of the BOI, and the angle of each vector indicates the preferred side of BOS for each orientation (cf. the symbols around the plot). The square orientations with a large BOI form a contiguous region in visual space, which is similar to BOS neurons in the primate visual cortex (e.g., Figure 2A, right).^3^ Filled symbols in Figure 2A indicate orientations for which BOI is significant (permutation test, see STAR Methods). The angular span between object locations at the preferred side of BOS for different border orientations (only orientations for which BOI is significant are considered) is referred to as the BOS span. For example, the span for the neuron shown in Figure 2A (left panel) is 144°. A substantial number of BOS units in PredNet have a large span, extending to ∼150°, similar to BOS neurons in the brain (Figure 2B; Figure S9A).

Next, we examined BOS tuning for different square positions and sizes. We set the orientation at that for which |BOI| was maximal, and then varied position (the position of the center of the central border varied along a line orthogonal to the border’s orientation; Figure 2C). We also varied square size for the central position (Figure 2D). We find that the response difference between scenes with opposite BOS was consistent for different positions or sizes in the population of BOS units in PredNet (Figures 2C and 2D, left panels), just like for BOS neurons in the brain (Figures 2C and 2D, right panels).^1^ We quantified this consistency by averaging BOI across different conditions (i.e., size or position, see STAR Methods). For all modules with a substantial number of BOS units (over 10 units), these averaged BOI values are statistically significantly positive (i.e., consistent with the tuning in the baseline condition; Figures S9B and S9C, bootstrapping test, see STAR Methods). Taken together, we find that border ownership signals in PredNet are robust to differences in square orientation, size and position, i.e., remarkably similar to BOS neurons in the primate visual cortex.^1^^,^^3^

### Object fragments on the non-preferred side suppress BOS units more than those on the preferred side

Neurophysiological experiments found that isolated object fragments in the surround modulate the activity of BOS neurons in a way that is consistent with BOS tuning: fragments on the non-preferred side of BOS suppress the response significantly more than fragments on the preferred side, which often have an enhancing effect.^33^ These modulatory effects were only significant in the presence of a border in the cRF. We analyzed how fragments in the surround modulated the activity of BOS units in PredNet.

Similar to Zhang et al. 2010,^33^ we divided a square object into 8 fragments: one center edge (CE) located at the image center, and 7 contextual fragments (two near corners [NC1 and NC2], two near edges [NE], two far corners [FC], and one far edge [FE]). This allows us to create two types of fragment scenes. The first type pairs one of the fragments in the surround with the CE (“with-CE”). Two additional scenes contain respectively only the CE, or all the fragments. The second type are identical scenes but without the CE (“without-CE”). For with-CE scenes, we defined the surround influence of a fragment as the unit’s response to the combination of that fragment and the CE, subtracted by the response to the CE-only scene (see STAR Methods). For without-CE scenes, the surround influence of a fragment was measured as the response to a scene with that fragment, subtracted by the response to a full-gray scene. Figure 2E displays the data for one example BOS unit in PredNet. First, we noticed that the absolute value of surround influence in with-CE scenes is larger than in without-CE scenes. This was the case for each PredNet module with at least 10 BOS units (Figure 2F for $E_{2}$ and other modules in Figure S10), and also in the population of all BOS units (Figure S10). This is similar to BOS neurons in the visual cortex (Figures 2G and 2H).^33^ Second, we compared the modulation effect between fragments on the preferred side and the non-preferred side. The preferred and non-preferred sides were determined solely from the responses to standard square scenes (Figure 1A). Despite this, we found that for all modules with more than 10 BOS units, the surround influence for most fragments is significantly more negative when they are presented on the non-preferred side compared to the preferred side (Figure 2F; and the same is true in the population of units across modules; Figure S10). This is also true for BOS neurons in the visual cortex (Figures 2G and 2H). These data support that BOS tuning in PredNet does not result from a single hotspot in the surround, but that multiple fragments collectively contribute, as is the case for BOS neurons in the brain.^33^

Note that these data also reveal differences between BOS units in PredNet and neurons in the brain. Despite the differences in amplitude, fragment modulation effects in PredNet often have the same sign on both sides (Figure 2F; Figure S10, although see module $E_{3}$ in Figure S10). This is different in the brain, where the modulation at the preferred side is (weakly) excitatory and the non-preferred side is (strongly) inhibitory (Figure 2H)^33^ This difference might be due to the fact that PredNet does not contain explicit categories of excitatory and inhibitory units, as opposed to the brain and some other ANNs.^34^ Inhibitory neurons may mediate the competition between neurons with opposite preferred sides of ownership in the brain.^35^

### BOS units exhibit hysteresis

**Figure 3:**
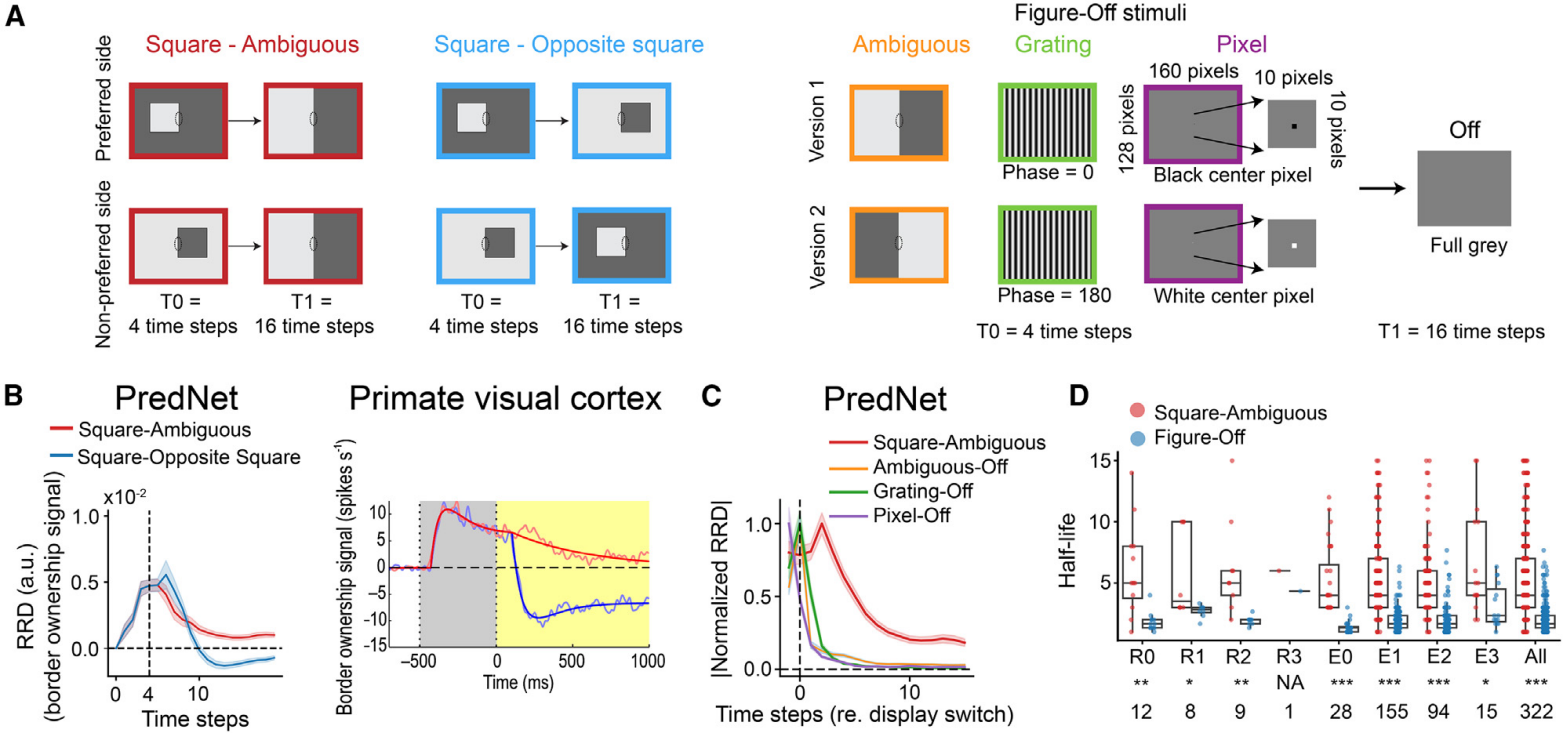
BOS signals in PredNet exhibit hysteresis, similar to BOS neurons in the brain (A) Three sequences with scene changes were used: squares transitioning to ambiguous borders (Square-Ambiguous), squares transitioning to squares with opposite border ownership (Square-Opposite Square), simple scenes (ambiguous, grating, or pixel) transitioning to a full gray scene (Figure-Off). Stimuli were presented at the orientation for which |BOI| was maximal. (B) The relative response difference (RRD, see STAR Methods) represents the difference in response between scene sequences that start with a square on the preferred and the non-preferred side (for Square-Ambiguous or Square-Opposite Square), or between version 1 and version 2 (for Figure-Off). Left: RRD of BOS units from PredNet ($E_{2}$ module, n = 132 units). Line and error bands represent the mean and SEM. Right: BOS signal for BOS neurons in the brain to similar sequences (reproduced from O’Herron and von der Heydt [2009],^24^ with permission from Elsevier). (C) Mean and SEM of the absolute value of the normalized RRD (normalized to maximal value) across BOS units in the $E_{2}$ module for different sequences (*n* = 132 units). (D) Half-life is defined as the number of time steps after which RRD is reduced to half of its maximum. Each dot corresponds to one BOS unit. Figure-Off data show the average across the three subtypes shown in (A). Only units for which half-life was defined for all conditions were included in this panel (see STAR Methods). All: units concatenated from all modules. Numbers at the bottom indicate the number of included units per module. Asterisks indicate the statistical significance of the difference in half-life between Square-Ambiguous and Figure-Off: NA: not applicable; ∗∗∗: *p* < 0.001; ∗∗: *p* < 0.01; ∗: *p* < 0.05 (Wilcoxon signed-rank test). See also Figure S11. Boxes indicate the interquartile range between the first and third quartiles with the central mark inside each box indicating the median. Whiskers extend to the lowest and highest values within 1.5 times the interquartile range from the box boundaries.

A remarkable characteristic of BOS neurons in the brain is that the BOS signal persists for hundreds of milliseconds, even when the contextual information that defines the side of ownership disappears.^24^^,^^25^ We tested if BOS units in PredNet also exhibit this phenomenon. We used a Square-Ambiguous Sequence similar to what was used in physiology experiments.^24^ The sequence consists of a full square scene (i.e., similar as in Figure 1A) in the first four time steps, which transitions into a scene with a border that is ambiguous for border ownership (Figure 3A, left; presented during sixteen time steps). We presented these sequences to PredNet and computed the time course of the relative response difference (RRD), defined as the difference in response between the preferred and non-preferred square sides, normalized by the average response (see STAR Methods). The RRD for BOS units remains positive for multiple time steps (Figure 3B left, red function; Figure S11). The units thus respond differently to the ambiguous scene (which is identical in the two sequences), depending on stimulus history, a phenomenon called hysteresis. BOS neurons in macaque visual cortex show a similar hysteresis (Figure 3B right, red function).^24^

To determine whether this persistent BOS signal is longer than the typical signal decay, we analyzed the response for two control sequences. The first is Square-Opposite Square, which starts with a full square and then switches to another full square image with opposite border ownership (and opposite luminance, so that the contrast polarity of the central border remains the same; Figure 3A, middle). The RRD for this sequence decays much faster and stabilizes at a negative value, reflecting the switch in BOS (Figure 3B, left, blue function; Figure S11A). Again the same pattern occurs in BOS neurons in the brain (Figure 3B, right, blue function).^24^^,^^25^ To analyze how these patterns compare with the general signal decay in these same units, we also analyzed responses to non-BOS stimuli that were simply turned off after four time steps (“Figure-Off”). In these sequences, a simple scene (three subtypes: ambiguous, grating, or pixel) is followed by a full gray scene (Figure 3A, right). Again the RRD of PredNet’s BOS units decays faster to these sequences than to the Square-Ambiguous Sequence (Figures 3C and 3D; Figure S11B). The RRD half-life is significantly longer for Square-Ambiguous Sequences than for Figure-Off sequences (Figure 3D). The persistent signal is thus specific for the BOS sequence and not a general feature of these units. Together, we find that BOS signals in PredNet have similar dynamic characteristics as BOS neurons in the brain: the BOS signal persists when contextual information disappears such that the side of BOS becomes ambiguous, but quickly updates when the context indicates a switch in BOS.

### BOS units contribute more to prediction than non-BOS units for videos with moving objects

Our data presented thus far demonstrate that units with brain-like tuning for BOS exist in PredNet, a network trained to predict future visual input in video sequences. This suggests that BOS units specifically aid in predicting future video frames. To test that, we conducted ablation experiments in PredNet. In each of these experiments we ablated a subset of either BOS units or non-BOS units (i.e., candidate units that did not pass the criterion for BOS-selectivity, see STAR Methods), by freezing their activity at 0 (baseline activity), resulting in no output currents. This is functionally identical to removing those units from the network. We presented translating-square videos (40 unique videos in which a square moves at a constant velocity, Figure S12, top) to PredNet. We measured the prediction performance of PredNet to these videos, both before and after ablation.

**Figure 4:**
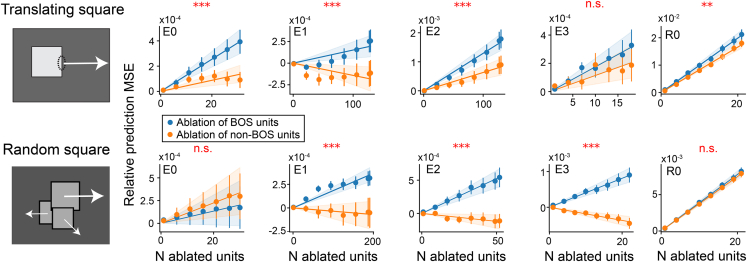
Ablating BOS units in PredNet increases prediction error more than ablating non-BOS units for videos with moving objects The left shows an example frame of each video type (arrows indicate motion and are not part of the frame). Translating square videos show a square moving at constant speed; random square videos show a random number of squares of different sizes, initialized at random positions and moving at random, constant velocities (see also Figure S12). Right panel shows the relative prediction mean squared error (RPE) for different numbers of ablated units. RPE measures the relative change of prediction error due to ablation. Non-BOS units are candidate units that do not pass the criterion of BOS selectivity. Dots and error bars denote respectively the mean and SEM of the RPE across 10 randomly chosen video samples. The RPE of one video sample is the average RPE of 10 samples of unit ablation (STAR Methods). The solid line indicates the best linear fit, with bands indicating the 95% confidence interval. The red text above the panels indicates whether the slopes of the lines differed significantly between BOS- and non-BOS-unit ablation. ∗∗∗: *p* < 0.001; ∗∗: *p* < 0.01; n.s.: not significant (bootstrapping test). Modules $R_{1}$, $R_{2}$, and $R_{3}$ contain a small number of valid BOS/non-BOS units for ablation (see STAR Methods and Figure S14) and are therefore not included in this analysis. See also Figures S12–S15.

The impact of unit ablation on video prediction is shown in Figure S13 (top row). Here, we introduce the metric “relative prediction mean squared error (RPE),” defined as the normalized difference (post- vs. pre-ablation) of the mean squared prediction error (see STAR Methods). A positive RPE represents an increase in prediction error after ablation. To quantify the overall effect of ablation in each module, we measured the slope of the relation between RPE and number of ablated units using linear regression, and a bootstrapping test to assess the statistical significance of this slope between ablating BOS units or non-BOS units (indicated with red symbols in Figure S13, top row). We find that the RPE is significantly higher when BOS units were ablated than when non-BOS units were ablated for most modules. We wondered if this could be explained by a difference in responsiveness: BOS units may respond more to these video frames than non-BOS units. To explore that possibility, we subsampled the populations to ensure there were no statistically significant differences in response magnitude to the videos (Wilcoxon rank-sum test, *p* > 0.5, see Figure S14 and STAR Methods). The ablation experiment in these subsampled populations shows the same pattern, ruling out that the RPE difference is due to a difference in average response (Figure 4, top row). The data thus indicate that BOS units contribute more than non-BOS units in predicting future frames for these videos.

We wondered if BOS units also contribute to prediction of videos with multiple objects. We generated videos with several squares that were randomly positioned and moved in random directions (Figure S12, middle). When we performed the same ablation experiment for these videos, we find the same pattern: BOS units typically contribute more to prediction than non-BOS units, even though, again, PredNet was not exposed to such videos during training (Figure 4, bottom).

Finally, we wondered if BOS units aid in prediction with any video. We performed the same experiment in a set of 41 natural videos from the KITTI database^30^ (Figure S12, bottom). This is the same database that was used to train PredNet, but we only included videos that were not used during training. BOS units in the $E_{2}$ module contribute more to prediction than non-BOS units (Figure S13). Note that these videos are much higher dimensional than the translating square and random square videos, and there is a high degree of heterogeneity within the small set of 41 videos. This results in smaller overall RPEs when averaged across videos than for the square videos, and not enough statistical power to precisely estimate RPE in the subsets of units with similar responsiveness (Figure S15).

Together these experiments suggest that BOS units emerge in PredNet because they contribute more to prediction than non-BOS units for videos with moving objects.

## Discussion

The assignment of borders to foreground surfaces is thought to be a key step in visual scene segmentation,^13^^,^^36^ and a substantial fraction of neurons in visual areas V2 and V4 of the primate brain signal this ownership of local borders.^1^^,^^2^^,^^3^ It is poorly understood why the brain resorts to using this particular representation. Here we discovered that units selective for BOS also emerge in an ANN, PredNet,^26^ that was trained to predict future input in natural videos. Importantly, the network was not explicitly trained to distinguish foreground from background or to identify objects in visual scenes. Interestingly, BOS units in PredNet share several properties with BOS neurons in the brain (robustness for different positions, orientations, and sizes^1^^,^^3^; asymmetric functional effects of object fragments on opposite sides of the border^33^; and BOS hysteresis^24^), suggesting that these signals are functionally similar to those in the brain. Finally, we found that ablation of BOS units affect prediction accuracy more than ablation of non-BOS units. Overall, our results suggest that BOS units might emerge in neural networks trained on natural, complex dynamic input primarily because they are particularly helpful to efficiently process such input, even if segmentation is not required.

That BOS neurons are important under dynamic conditions is consistent with the phenomenon of BOS signal hysteresis that was discovered in the brain^24^^,^^37^: BOS signals persist when borders become ambiguous (contextual information disappears; red trace in Figure 3B right panel), but quickly update when contextual information becomes inconsistent with the current BOS signal (blue trace in Figure 3B right panel). This suggests that these neurons make use of dynamic aspects of the visual input to infer the most likely organization of object surfaces in visual scenes (scene segmentation). Because different parts of the same object surface tend to move coherently in natural environments, scene segmentation might be helpful to predict future visual input. The present data support indeed a tight link between prediction and BOS signals in neural networks. PredNet’s architecture was inspired by the predictive coding framework. This theory proposes that a major function of the sensory cortex is to predict incoming sensory stimuli.^38^^,^^39^^,^^40^^,^^41^^,^^42^ The hierarchical organization of visual cortical areas is proposed to compute an internal model of the external world, and feedback from areas higher in the hierarchy (e.g., V4 and IT) is thought to reflect predictions from this internal model, which is then compared with incoming sensory information in lower areas (e.g., V1 and V2).^38^ Our findings indicate that an architecture inspired by predictive coding can lead to BOS signals, with properties very similar to those in the brain, even without explicitly training the network to localize or identify objects in visual scenes.

Our finding of brain-like BOS signals in PredNet, a recurrent ANN, is consistent with the notion that feedback is important in BOS computations (reviewed by von der Heydt^13^). Even though BOS neurons in the brain signal BOS within less than 100 ms after stimulus onset (thus within the interval between adjacent frames in the videos used to train PredNet^1^^,^^3^^,^^43^), the prevailing physiological evidence indicates that these cells rely on top-down input to compute BOS.^13^^,^^44^^,^^45^^,^^46^ Because of the short latency, this feedback cannot originate from the highest level in the hierarchy. Instead it may come (in part) from area V4, which has been proposed to contain grouping cells that compute proto-object representations with short latency, i.e., an early prediction of the shape and location of objects in the scene, and/or from dorsal stream areas.^45^ Such feedback could explain border ownership signals in lower areas,^6^^,^^13^^,^^33^^,^^47^ and a recent study indeed found evidence that supports the existence of grouping cells in V4.^48^ Response dynamics and laminar organization of BOS neurons align better with feedback models than with alternatives that solely rely on intra-areal horizontal connections or feedforward connections.^3^^,^^4^^,^^33^^,^^49^^,^^50^^,^^51^^,^^52^^,^^53^ Consistent with the importance of recurrence for BOS computations, a previous study found that BOS signals in a feedforward ANN trained on scene segmentation differed from those in the brain in several ways.^16^ For example, analysis of the receptive field of such units suggested that their BOS selectivity depended on differences within the cRF, thus, in contrast to BOS neurons in the brain and the BOS units in PredNet that we report here. Feedforward networks thus seem to lack the essential mechanisms to provide the required extra-classical RF contextual information.

A prior study showed that PredNet units signal illusory contours and end-stopping.^18^ The emergence of BOS signals as well as these other extra-cRF phenomena under the predictive coding framework raises a question: do these phenomena result from a single hierarchical neural computation? Several lines of prior research are consistent with that possibility.^18^^,^^32^^,^^38^^,^^39^^,^^41^^,^^54^ A complete answer to this question is hard to obtain by solely doing physiology experiments: detailed maps of neural connections are often unavailable, and it is challenging to precisely manipulate these connections. ANNs have the unique advantage of possessing complete connection profiles,^23^^,^^55^^,^^56^ and allow one to perform ablation studies. Our work thus establishes PredNet as a useful complementary tool toward achieving an understanding of these computations.

PredNet’s E modules have been hypothesized as being akin to superficial layers (L1/2/3), and the R modules as akin to deeper layers (L5/6) of the visual cortex, following the proposed functional specialization of cortical layers in predictive coding.^18^^,^^27^^,^^38^ The presence of BOS signals in both E and R modules is intriguing. In area V4, BOS neurons have been found in superficial, granular, and deep laminar compartments.^3^ However, equating different modules in PredNet with cortical layers is too simplistic. For example, units in the R modules have lateral connections (ConvLSTM), whereas those in the E modules lack these, unlike in physiology where lateral connections exist in both superficial and deep compartments.^41^^,^^57^^,^^58^ Further studies are needed to understand the functional role of different areas and layers in this hierarchical computation and the communication between them, and the similarities and differences between such modules in the brain and ANNs.^18^^,^^27^ Another interesting observation is that many BOS-units occur in PredNet’s E-modules, thus representing prediction errors of the current video frame that then inform better predictions over time. Indeed, ablation of these units in individual E-modules worsens predictions (Figure 4). BOS signals in the brain might thus, in part, also represent prediction errors.

Our discovery of BOS units in PredNet and the ablation experiments indicate that BOS neurons may be useful for video prediction. To predict future visual input, it is useful to predict object motion.^59^ Objects typically move as a whole, i.e., pixels within object boundaries most likely move together.^60^ Because BOS units indicate which pixels belong to an object surface, they may help to predict by allowing the system to easily apply a uniform optical flow to objects. Indeed, in computer science, incorporating optical flow,^61^^,^^62^ disentangling object motion from content,^63^^,^^64^^,^^65^^,^^66^ and separating foreground objects from background^60^^,^^67^ have been shown to improve video prediction performance. Beyond video prediction, in object recognition, deep neural networks have been criticized for relying mostly on textural information to recognize object categories rather than on object shapes,^68^^,^^69^^,^^70^ in contrast to human visual perception^71^^,^^72^ (but perhaps more akin to mouse visual perception^73^). Explicitly embedding a BOS unit module may guide neural networks to rely more on shapes, and potentially achieve more robust recognition as well as prediction.

Overall, our work demonstrates that brain-like BOS signals emerge in a self-supervised network trained to predict future input. This implies a shift from the traditional view of BOS as a static “what stream” operation toward a computation that is highly beneficial to predict future input in natural dynamic environments.

### Limitations of the study

While our results demonstrate that a recurrent deep neural network trained on video prediction develops BOS signals that parallel many properties of BOS neurons in the primate visual cortex, there are several limitations of the study. First, our data do not allow to infer if this parallel extends to dimensions of the sensory input that we could not easily explore with PredNet, such as depth^7^ or eye movements.^25^ Second, PredNet’s architecture differs from the brain in several ways, such as the lack of an explicit separation between inhibitory and excitatory neurons,^34^ which may have specific roles in this computation in the brain.^35^ Third, while this study demonstrates the similarity between an ANN and the brain at the representational level (i.e., neural responses), it does not provide a mechanistic explanation for how BOS signals contribute to video prediction. Future work is needed to address these gaps and further elucidate the computational principles underlying BOS signals and their role in visual processing.

## Resource availability

### Lead contact

Further information and requests for resources and reagents should be directed to and will be fulfilled by the lead contact, Tom P. Franken (ftom@wustl.edu).

### Materials availability

This study did not generate new reagents.

### Data and code availability

• All data reported in this paper will be shared by the lead contact upon request. We have also used the existing, publicly available pre-processed KITTI data that have been deposited by Lotter et al. (2017),^26^ accessible at https://www.dropbox.com/s/rpwlnn6j39jjme4/kitti_data.zip.
• The original code to analyze border ownership responses in PredNet has been deposited at Figshare and is publicly available at https://doi.org/10.6084/m9.figshare.28533842 as of the date of publication.
• Any additional information required to reanalyze the data reported in this paper is available from the lead contact upon request.

## Acknowledgments

This project was supported by 10.13039/100000002NIH grants R00EY031795 (to T.P.F.) and R21EY036566 (to T.P.F. and R.W.), funding from the OVCR Seed Grant Program at Washington University (to T.P.F. and R.W.), a NARSAD Young Investigator Grant from the Brain & Behavior Research Foundation (to T.P.F.), and funding from the Incubator for Transdisciplinary Futures (to R.W., cluster *Toward a Synergy Between Artificial Intelligence and Neuroscience*).

## Author contributions

Conceptualization, Z.Y., R.W., and T.P.F.; methodology, Z.Y., R.W., and T.P.F.; investigation, Z.Y.; supervision, R.W. and T.P.F.; writing, Z.Y., R.W., and T.P.F.

## Declaration of interests

The authors declare no competing interests.

## Declaration of generative AI and AI-assisted technologies in the writing process

During the preparation of this work the authors used ChatGPT-4o in order to improve the readability of the manuscript. After using this tool, the authors reviewed and edited the content as needed and take full responsibility for the content of the published article.

## STAR★Methods

### Key resources table

**Table:** 

REAGENT or RESOURCE	SOURCE	IDENTIFIER
**Deposited data**		

Preprocessed KITTI Data	Lotter et al.^26^	https://www.dropbox.com/s/rpwlnn6j39jjme4/kitti_data.zip

**Software and algorithms**		

Analysis code used for this study	This study	https://doi.org/10.6084/m9.figshare.28533842
PredNet model code	Lotter et al.^26^	https://github.com/coxlab/prednet
Python	Python Software Foundation	https://www.python.org/
Tensorflow	Abadi et al.^74^	https://www.tensorflow.org/
numpy	Harris et al.^75^	https://numpy.org/
scipy	Virtanen et al.^76^	https://scipy.org/
scikit-learn	Pedregosa et al.^77^	https://scikit-learn.org/stable/
Matplotlib	Hunter^78^	https://matplotlib.org/
pandas	Reback et al.^79^	https://pandas.pydata.org/
Statsmodels	Seabold et al.^80^	https://www.statsmodels.org/stable/

### Method details

#### PredNet architecture and training

In this study, we utilized the ANN PredNet, which was developed and trained by Lotter et al. (2017)^26^ (code is available at: https://github.com/coxlab/prednet). Here we briefly summarize PredNet’s architecture and how it was trained. PredNet is an ANN that has four layers (labeled as ‘l'). Each layer consists of four types of modules: the Representation module ($R_{l}$), the Prediction module (${\hat{A}}_{l}$), the Prediction target module ($A_{l}$), and the Prediction Error module ($E_{l}$). Updating unit activities in PredNet involves two main stages at each time step:

Top-to-Bottom Update: The network updates the R modules from top to bottom at each time step. Each $R_{l}$ module gets inputs from the $R_{l+1}$ module and the $E_{l}$ module. This updating process goes from the $R_{3}$ module to the $R_{0}$ module in sequence. The $R_{0}$ module then generates a predicted current video frame (${\hat{A}}_{0}$).

Bottom-to-Top Update and Error Calculation: The update process then reverses, proceeding from bottom to top. The network calculates the prediction error by comparing ${\hat{A}}_{0}$ with the actual next video frame, $A_{0}$. This error is bifurcated into positive and negative parts (akin to biological ON-center and OFF-center neurons). Positive and negative errors are grouped in the $E_{0}$ module. $E_{0}$ then outputs a target prediction $A_{1}$, which gets compared with ${\hat{A}}_{1}$ produced from $R_{1}$. The error from this comparison is computed in the $E_{1}$ module. The network continues this process up to the final layer (layer 3).

Mathematically, the PredNet dynamics are defined by

(Equation 1) $$A_{l}^{t}=\left\{ \begin{array}{cc} x_{t}, & \text{if}\;l=0\\ \text{MAXPOOL}\left(\text{RELU}\left(\text{CONV}\left(E_{l-1}^{t}\right)\right)\right), & l>0 \end{array}\right.\;\hat{A}{\;}_{l}^{t}=\text{RELU}\left(\text{CONV}\left(R_{t}\right)\right)\;E_{l}^{t}=\left[\text{RELU}\left(A_{l}^{t}-\hat{A}{\;}_{l}^{t}\right);\text{RELU}\left(\hat{A}{\;}_{l}^{t}-A_{l}^{t}\right)\right]\;R_{l}^{t}=\text{CONVLSTM}\left(E_{l}^{t-1},R_{l}^{t-1},\text{UPSAMPLE}\left(R_{l+1}^{t}\right)\right)$$

where t is the time step, $x_{t}$ is the actual video frame. ConvLSTM uses a tanh activation function, which means that the R module activity can be negative (possible values range from −1 to 1). Because biological neurons do not have negative spike rate, PredNet unit’s response was measured in this study as the unit activation plus one, i.e., the response baseline was shifted by +1 in all modules (after PredNet’s computation was completed, thus this did not affect PredNet’s algorithm). The PredNet architecture contains 3, 48, 96, and 192 convolution channels in layers 0 to 3, respectively. The input image size is 128 by 160 pixels. The number of units in R modules are respectively 61,440 in $R_{0}$, 245,760 in $R_{1}$, 122,880 in $R_{2}$, and 61,440 in $R_{3}$. The $A_{l}$ and ${\hat{A}}_{l}$ modules have the same number of units as the $R_{l}$ module. Due to the bifurcation of positive and negative error, E modules have twice the number of units compared to the R modules.

The training loss function is applied on the prediction error

(Equation 2) $$L_{train}=\frac{1}{N_{t}N_{0}}\sum_{t}\;\sum_{n_{0}}\;E_{0}^{t}$$

where $N_{t}$ is the number of time steps used in training, $N_{0}$ is the number of $E_{0}$ units. The training utilized the KITTI dataset, which contains videos recorded from car-mounted cameras in Germany. Videos were segmented into sequences of 10 continuous frames. These frames were then center-cropped and downscaled to a resolution of 128 by 160 pixels. Overall, this preprocessing procedure yielded 4212 video sequences. These sequences were split into training, test, and validation datasets of 4114, 83, and 15 video sequences respectively. The training dataset was used to optimize PredNet through backpropagation with the Adam optimizer, using the loss function described above (Equation 2). After training, all weights were frozen.

In one control analysis (Figure S8), we trained an untrained instance of PredNet on static stimuli, i.e., sequences in which all images were identical. These sequences were generated from the same KITTI training videos used above. Each frame of the 4114 training videos was duplicated 10 times along the temporal axis, forming 41140 sequences of 10 identical images in total. These sequences were then used to train an untrained instance of PredNet to do “next-frame prediction”. An example network prediction is shown in Figure S8A and BOS unit analyses of this network are shown in Figure S8. The code used to analyze the trained PredNet was implemented in Python along with Python packages^74^^,^^75^^,^^76^^,^^77^^,^^78^^,^^79^^,^^80^ and is publicly available (https://github.com/AgeYY/BOS-in-Video-Prediction).

#### Stimulus presentation

PredNet accepts sequences of images where each image of the sequence is successively fed into the network (one image per time step). In some of our analyses, we used static stimulus presentations (Figures 1C–1E and 2A–2F). In those cases, an identical image was used during multiple sequential time steps such that it was continuously presented to the network. In other analyses, we used dynamic image sequences (Figure 4), in which each successive image corresponded to a successive video frame in the stimulus (see also examples in Figure S12). Finally, there were biphasic, ‘switching’, stimulus sequences (Figure 3). These consisted of a sequence of two different images, each of which was fed into the network continuously during a specific number of time steps, as indicated in Figure 3.

#### Receptive field mapping

We measured the cRF of units in PredNet empirically, using sparse noise stimuli (Figure S1), similar to the approach used in physiology. The recurrent features of PredNet (ConvLSTM units^81^^,^^82^ and feedback connections^26^) impede a straightforward analytical computation of the cRFs. We created an image (128 x 160 pixels) with one pixel set to either white or black, while all others were set to gray (gray level = 0.5, scales from 0 to 1). 40,960 (128 × 160 × 2 where the factor two is for black and white pixel) unique images (128 x 160) were generated, each featuring a distinct single pixel, in either white or black. These images were repeated for four time steps, yielding a total of 40,960 sequences. For each unit, recorded activity to these sequences was summarized into two heatmaps (each size 128 × 160), each representing responses to respectively white-pixel and black-pixel scenes. For example, the white heatmap’s i,j entry is the single unit’s time-averaged response to a scene with a white pixel located at i,j (gray otherwise).

The two heatmaps (for one unit) were then z-scored and converted to absolute values. These heatmaps were merged into one heatmap by taking the maximum absolute values for each entry. This merged heatmap summarizes the unit’s maximum response to a pixel at each location irrespective of its color (white or black). The cRF for each unit was defined as the union of the pixel positions for which the absolute values of the maximal z-scores across both heatmaps exceed 1.

CRF diameter was measured as follows. We computed the distance between every pixel in the cRF and the cRF center (defined as the mean position of all pixels in the cRF). The diameter of the cRF was measured by doubling the maximum of these distances.

#### Standard square scenes

Scenes with square objects are commonly used in neurophysiological studies to assess whether a unit is selective for BOS^1^^,^^2^^,^^3^^,^^48^ and this selectivity is known to extend to natural images.^5^ We used similar scenes, consisting of a square with a size (width) of 50 pixels, positioned with one border centered at the center of the scene (central border). The color of the square and the background can be either light (gray level = 0.33 on a scale from 0 to 1) or dark gray (gray level = 0.66), but they are always different from each other in a given scene. These square scenes can be defined mathematically by three parameters. The first parameter, α, denotes the square’s orientation, with a range from 0 to 180°. The second parameter, β, is a binary variable indicating which side the square is given a fixed orientation (i.e., side of ownership). The final parameter, γ, is a binary variable that indicates the contrast polarity across the central border. For each square orientation defined by α, there are four possible square scenes, determined by different combinations of β and γ. Each unique scene was fed into the network continuously during 20 time steps. We used 10 different orientations (equally spaced by 18°).

#### Inclusion criteria

To define selectivity for border ownership, it is important to verify that the units under examination respond to changes in border ownership rather than to low-level stimulus changes within the cRF. Therefore, similar as in neurophysiology studies, we restricted our analysis to units that passed the following two criteria (termed ‘candidate units'). First, the unit’s cRF must include the center of the scene. Because the central border of the square scenes was placed exactly in the scene center, this ensured that the unit’s cRF includes the center of this border. Second, the cRF must fit within a circle centered at the center of the scene and with a radius of 20 pixels. Because the square size (width) is 50 pixels, this ensures that the cRF does not overlap with any other border of the square besides the central border.

#### Computing selectivity for border ownership

Similar to neurophysiology studies^1^^,^^2^^,^^3^ we quantified tuning for border ownership using the BOI. This is computed from the response of PredNet units to standard square scenes. The BOI is defined as

(Equation 3) $$BOI\left(\alpha \right)=\frac{Res\left(\alpha ,1,0\right)-Res\left(\alpha ,0,0\right)+Res\left(\alpha ,1,1\right)-Res\left(\alpha ,0,1\right)}{Res\left(\alpha ,1,0\right)+Res\left(\alpha ,0,0\right)+Res\left(\alpha ,1,1\right)+Res\left(\alpha ,0,1\right)}$$

where Res(α,β,γ) is the unit’s time-averaged (between 0 and 19 time steps) responses to a square scene specified by orientation α, side-of-ownership β and contrast polarity γ. The sign of the BOI thus indicates which side (β) of BOS (for a given orientation) the unit prefers, and the magnitude indicates the strength of the BOS tuning.

To evaluate the overall BOS selectivity across orientations, we defined $B_{av}$ as the circular average of the BOI across α. Similar to BOI, the magnitude of $B_{av}$ is a measure of the strength of BOS tuning, and its angle indicates the unit’s preferred side of BOS.

We evaluated the statistical significance of $B_{av}$ using a permutation test. In this test, we shuffled the labels that signified the side of BOS (β) for each orientation α. These data were then used to compute a shuffled BOI(α) and $B_{av}$. This procedure was repeated 5,000 times to generate a set of 5,000 $B_{av}$ values after shuffling, for each unit. Denoting the quantile of the unshuffled $B_{av}$ among the shuffled $B_{av}$ as Q, the *p*-value (two-tailed) was estimated as 2×min{Q,1−Q}. Units with a *p*-value less than 0.05 were defined as BOS units. 95% confidence intervals on the proportions of the units for which $B_{av}$ was significant were computed using the Wilson score.^83^

Note that the values of $B_{av}$ and BOI reported here cannot easily be compared with similar indices in neurophysiology, because these values change when the DC level of unit activity is changed. As mentioned above, to avoid negative values for unit activity in PredNet, we arbitrarily increased activity levels by +1. Furthermore, the average BOI across time depends on when the response starts relative to the duration of the analysis window. This is at ∼50% of the window duration for the unit shown in Figure 1D (top), whereas in physiology studies this is typically closer to ∼10%. For example, the activity functions shown in Figure 1D (top panel) show a BOI of 0.0149 at time step 10, but computing this without adjusting the unit activation (i.e., without +1) leads to BOI = 0.68. Zhou et al. use ‘response ratio’ to quantify the magnitude of BOS tuning, defined as the ratio of the mean response to non-preferred BOS over the mean response to preferred BOS. For the activity functions shown in Figure 1D (top panel) this value is 0.561 (averaged across analysis window), well within the range of values found for neurons in the macaque visual cortex.^1^

#### Variations of square scenes

In these experiments, varied parameters were square orientation (α), side-of-ownership (β), contrast polarity (γ), position along the orientation (d), and size (s). We first measured the response to a set of four standard square scenes (Figure 1A). For each unit, the orientation α is fixed at the orientation with the maximum |BOI|. The position is zero, indicating that the square border intersects exactly with the scene center, and the square size (width) is 50 pixels. BOS units’ responses were averaged over time and contrast polarity. The β value with the larger averaged unit response was defined as the preferred side ($\beta_{p}$), whereas the opposite was defined as the non-preferred side ($\beta_{np}$). These preferences were solely determined by the standard square scenes.

We then examined the effect of changing square size. All other parameters remained the same as in the standard square scenes stated above, except for square size. Eight square sizes were used, ranging from 10 to 80 pixels. For each unit i and each square size $s_{j}$, we computed the responses averaged across time and contrast polarity, yielding ${\bar{r}}_{i,j}\left(\beta_{p}\right)$, ${\bar{r}}_{i,j}\left(\beta_{np}\right)$. We then normalized two response arrays of each unit i: ${\tilde{r}}_{i,j}\left(\beta \right)={\bar{r}}_{i,j}\left(\beta \right)/\sum_{j}{\bar{r}}_{i,j}\left(\beta \right)$, where β can be $\beta_{p}$ or $\beta_{np}$. Figure 2D (left panel) displays ${\tilde{r}}_{i,j}\left(\beta \right)$ across units i. For each unit i and square size $s_{j}$, we computed a BOI as the difference in response between the $\beta_{p}$ and $\beta_{np}$, i.e., $BOI_{i,j}=\left({\tilde{r}}_{i,j}\left(\beta_{p}\right)-{\tilde{r}}_{i,j}\left(\beta_{np}\right)\right)/\left({\tilde{r}}_{i,j}\left(\beta_{p}\right)+{\tilde{r}}_{i,j}\;\left(\beta_{np}\right)\right)$. We performed a bootstrapping test to assess statistical significance of this metric. A BOI dataset consisted of $D^{BOI}=\left\{BOI_{i,j}\right\}$ for all units i and square sizes $s_{j}$. We obtained 10,000 bootstrap samples $D^{s}$ from this dataset. For each $D^{s}$, we computed an averaged BOI, denoted as ${BOI}^{s}$. The *p*-value was estimated as p=Q, where Q is defined as the quantile of 0 among all ${BOI}^{s}$. If *p*-value was smaller than 0.05, we concluded that the BOI averaged across size was statistically significantly positive in the population of BOS units (Figure S9).

The same procedures apply to varying square position, simply replacing square size with square position (Figure S9). Fifteen square positions were used, ranging from −30 to 26 pixels.

When examining the unit’s response to different orientations, we created square scenes with 10 possible orientations (equally spaced between 0 and 180°), keeping the position at 0 and size at 50 pixels. Units' responses were collected to compute the BOI for each orientation using Equation 3. Data from one example unit is shown in Figure 2A (left panel). To evaluate the statistical significance of BOI for a given orientation, we compared the unshuffled BOI to that in a null distribution. Unlike biological neurons, which differ in response from trial to trial, PredNet does not have noise. In order to obtain sufficient data to generate a shuffled distribution, for each orientation, we varied square size (10 different sizes were considered, conceptually mimicking 10 “repeated trials”). The unshuffled BOI for a given orientation was computed for this orientation across square size. The null distribution for BOI distribution was obtained by shuffling the labels indicating the side-of-ownership β (i.e., border ownership), separately for each square size and contrast polarity (5,000 shuffles). The quantile (Q) of the unshuffled BOI within shuffled BOI set was computed. The *p*-value (two-tailed) was estimated as 2×min{Q,1−Q}. If the *p*-value is less than 0.05, BOI along an orientation was said to be statistically significant (indicated in Figure 2A as filled circles). The above procedure resulted a subset of orientations with statistically significant BOIs. The span of each BOS unit was computed as the difference between the two most distant preferred object locations (circular distance between the two angles corresponding to those locations). 95% confidence intervals on proportions of units for the span smaller than a certain value were computed using the Wilson score.^83^

#### Square fragment scenes

##### Stimuli

The squares in the square scenes can be divided into eight fragments^33^: the centre edge (CE), which is the one in the middle of the scene; there are two near corners (NC), two near edges (NE), two far corners (FC), and one far edge (FE). To examine how these fragments modulate the activity of BOS units, the four standard square scenes (Figure 1A, orientation aligns with the preferred BOS orientation for each unit) were converted into fragmented square scenes, as described below.

To isolate one fragment, a 2D Gaussian filter (σ=5 pixels) was applied at the fragment’s center. This kept the fragment’s central region largely unaltered, while the parts of the scene further away gradually fade to a uniform gray (gray level = 0.5 on a scale from 0 to 1). For scenes with multiple fragments (e.g., ‘All’), a Gaussian filter was applied to each fragment. Note that the smallest distance between two fragment centers is 25 pixels, thus much larger than σ, resulting in negligible interference between filtered fragments at different locations.

Using this Gaussian filter method, we created 9 scenes with a Central Edge (‘with-CE’) and 9 scenes without a centre edge (‘without-CE’) for each of the four standard square stimuli (Figure 1A). Among the with-CE scenes, one scene only has the CE fragment, seven scenes have the CE and one additional fragment, and one scene has all fragments. The without-CE scenes are similar to the with-CE scenes, except that they do not contain the CE fragment. Thus the “all fragments” without-CE scene contains 7 fragments. Each unique scene was fed into the network continuously during 20 time steps.

##### Analysis

The NC fragment could potentially partially intersect with the cRF. To prevent this, we limited this analysis to the subset of BOS units whose cRFs fitted within a circle of 30 pixels diameter centered at the center of the scene. This more conservative selection yielded 30, 145, 71, 5 units from respectively E0 to E3, and 1, 3, 2, 0 units from respectively R0 to R3. For with-CE scenes, the surround influence of square fragment X is defined as the unit’s response to the X + CE scene subtracted by the response to the CE scene. Similarly, for without-CE scenes, the response to square fragment X is defined as the difference between the activity to scene X and that to a full gray scene. If X is FE, the surround influence of X is computed as above. Otherwise (X = FC, NE, NC), the surround influence of X is the average of the surround influences of two conjugate edges (e.g., CE1 and CE2).

The surround influences of X for all BOS units were computed, resulting in a list where the length equals the number of BOS units. To avoid bias in mean estimation due to outliers, outliers (1.5 interquartile range below the first quantile or above third quantile) were removed before computing the sample mean and SEM (Figure 2F; Figure S10). However, all units were included when performing statistical tests (indicated by figure caption). For within-module statistical tests, we only include modules with at least 10 BOS-units to decrease the chance of type II errors.

#### Assessing border ownership signal hysteresis

##### Stimuli

Each trial in the Square-Ambiguous sequences consisted of 20 time steps, broken down into two phases. Initially, Scene 0, one of the four standard square scenes (Figure 1A), was displayed during four time steps (T0 = 4). Subsequently, Scene 1 was shown during 16 time steps (T1 = 16). Scene 1 only contained a central border that divides the whole image into a left and a right half; hence the side of ownership of this border was ambiguous. The contrast polarity and orientation of Scene 1 were consistent with Scene 0 (i.e., the information in the cRF was the same).

Similarly, the Square-Opposite Square sequences started with one of the four standard square scenes as Scene 0. Scene 1 was a version of the square scene with reversed BOS, but maintaining contrast polarity for the central border. For example, if Scene 0 was panel 1 in Figure 1A, then Scene 1 was panel 2 in Figure 1A.

For Figure-Off Sequences, Scene 1 was always a full gray. Scene 0 depended on the subtypes: Ambiguous-Off, Grating-Off, and Pixel-Off sequences. For Ambiguous-Off, Scene 0 was an ambiguous border. It had two versions that vary in contrast polarity. In Grating-Off sequences, Scene 0 was a grating with a 10-pixel spatial period, and it had two versions with grating phases of either 0 or 180°. For Pixel-Off sequences, Scene 0 was gray except for a single pixel at the center, which was either white or black corresponding to two versions.

All scenes were generated such that the orientation corresponds to that for which each unit’s |BOI| was maximal.

##### Analysis

The Relative Response Difference (RRD, see Figure 3) is (a−b)/(a+b), where a indicates the time-averaged response to preferred stimuli, and b indicates the time-averaged response to non-preferred stimuli. Which stimulus was preferred only depended on the averaged response to Scene 0.

RRD half-life was defined as the earliest time after the scene switch where the absolute value of RRD was less than half of its maximum. The half-life across the three types of Figure-Off sequences were averaged in Figure 3D. For this analysis, we only included units for which the half-life of all three types of Figure-Off sequences could be measured (cases for which RRD never dropped to half of its maximum within the analysis window were excluded). The number of such units per module are shown in Figure 3D. The Wilcoxon signed-rank test was used to compare half-life between Square-Ambiguous sequences and Figure-Off sequences.

#### Ablation experiment

##### Video stimuli

We generated three types of videos to evaluate PredNet’s prediction performance (examples shown in Figure S12). (1) Translating Square videos include a square that moves at a constant speed and direction. Square size is 50 pixels and oriented such that the central border had a vertical orientation (square gray level = 0.33 and background gray level = 0.66 on a scale from 0 to 1). The initial position and velocity of the square were chosen such that the square was always in the scene center in the 10^th^ frame. Forty translating square videos were created, corresponding to 40 evenly spaced moving directions (equally spaced between 0 and 360°). (2) Random Square videos: each of these videos featured a random number of squares (between 1 and 5). At the beginning of each video, each square’s central position was randomly set in the scene. The size of each square was also randomly chosen (between 10 and 50 pixels), and the x and y components of each square’s velocity were randomly set at a value between −2 and 2 pixels/frame. Forty random videos were generated. (3) KITTI testing videos: 41 video sequences from car-mounted cameras were used (the test set of KITTI videos, i.e., those that were not used to train PredNet^26^). These videos were not selected for content, but they often had objects (e.g., cars) in the frame center. For all video types, each video consisted of 20 frames.

##### Subsampling units to match response magnitude

Unit activity in response to the videos were squared and averaged across all videos and time steps for each video type, resulting in Mean Squared Response (MSR). For each module and video type, we have two sets of MSR, one for the BOS units and another for the non-BOS units, denoted as $D^{bos}=\left\{r_{0}^{bos},r_{1}^{bos},\ldots ,r_{n}^{bos}\right\}$ and $D^{non-bos}=\left\{r_{0}^{non-bos},r_{1}^{non-bos},\ldots ,r_{m}^{non-bos}\right\}$, respectively, where n and m representing the number of BOS and non-BOS units in one module.

For each of the $D^{bos}$ and $D^{non-bos}$, we subsampled k=min{n,m} units (1,000 samples). This resulted in 1,000 pairs of sampled datasets, denoted as $D_{s}^{bos}$ and $D_{s}^{non-bos}$, with s ranging from 1 to 1,000. For each pair, we computed a score to measure the similarity between datasets in a pair

(Equation 4) $$\phi_{s}={\left[mean\left(D_{s}^{bos}\right)-mean\left(D_{s}^{non-bos}\right)\right]}^{2}+{\left[median\left(D_{s}^{bos}\right)-median\left(D_{s}^{non-bos}\right)\right]}^{2}$$

where the mean(·) and median(·) represent those quantities of the dataset. The dataset pair with smallest score $\phi_{s}$ was subjected for further statistical analysis, using the Wilcoxon rank-sum test and the t-test. If both *p* values were larger than 0.5, we considered the dataset pair as our final subsampled datasets. If not, we reduced k by 1 and repeated the procedure above. This whole procedure ensures that both BOS and non-BOS populations have the same number of units (equal to k), and their MSRs do not show a significant difference. Figure S14 displays the MSR of the obtained subsampled unit populations.

##### Computing the prediction error

For each video type, we created $N_{\alpha }$ bootstrapped samples, each containing $N_{z}$ videos. We denoted $v_{z}^{\alpha }$ as the $z^{th}$ video in the $\alpha^{th}$ bootstrapped sample, with α ranging from 0 to $N_{\alpha }-1$, and z from 0 to $N_{z}-1$. In this study, $N_{\alpha }=N_{z}=10$.

For each video $v_{z}^{\alpha }$, we performed the ablation experiment several times, for different samples of ablated units, in each module separately. Units were ablated by freezing their activity at 0 (baseline) – this effectively removes those units from the network. We varied the number of ablated units n (ranges from 1 to $\min\left\{N_{bo},N_{non-bo}\right\}$, where $N_{bo}$ and $N_{non-bo}$ indicate respectively the number of BOS and non-BOS units available in the module). For each n, we generated $N_{u}=10$ bootstrapped unit samples from the unit pool (i.e., either from the BOS/non-BOS unit population in each module). A single sample is denoted as $u_{i}^{b,n}$ where b is a Boolean variable indicating whether the ablated units are BOS units or non-BOS units, and $i=1,2,\ldots ,N_{u}$ represents the $i^{th}$ unit sample. For each ablation sample $u_{i}^{b,n}$, the unit activity in the sample was set to zero. Mean-squared prediction error (MSE) was measured as the mean-squared difference between the predicted (${\hat{A}}_{0})$ and actual frames ($A_{0})$, averaging over all pixels and time steps. RPE of one video and one ablation sample was computed as

(Equation 5) $$RPE\left(v_{z}^{\alpha },u_{i}^{b,n}\right)\equiv {RPE}_{z,i}^{\alpha ,b,n}=\left[MSE\left(v_{z}^{\alpha },u_{i}^{b,n}\right)-MSE\left(v_{z}^{\alpha },0\right)\right]/MSE\left(v_{z}^{\alpha },0\right)$$

where $MSE\left(v_{z}^{\alpha },0\right)$ represents the MSE to the same video without ablation. We then computed the average RPE for a single video sample α and a given number of n ablated units:

(Equation 6) $${RPE}^{\alpha ,b,n}={\left\langle {RPE}_{z,i}^{\alpha ,b,n}\right\rangle }_{z,i}$$

where ${\left\langle \cdot \right\rangle }_{z,i}$ represents the average across indices z and i. Dots and error bars in Figure 4 show the mean and SEM of ${RPE}^{\alpha ,b,n}$ across different video samples α, with respect to the number of ablated units n, for the subsampled population (see above for how subsampling was done). Figure S13 shows the result for the original population (without subsampling).

We model the ${RPE}^{\alpha ,b,n}$ as a linear model

(Equation 7) $${RPE}^{\alpha ,b,n}=k^{b}n+\epsilon$$

where the intercept term is zero because the RPE is zero when no units are ablated. ϵ is an error term with a zero mean and a constant unknown variance, and $k^{b}$ is the slope of a line that represents the average change in RPE if one additional unit is ablated (b=bos for ablation of BOS units, b=non−bos for ablation of non-BOS units). We are interested in determining whether the slope $k^{bos}$ is significantly different from $k^{non-bos}$. A bootstrap method is used as follows.

Observations are denoted as $D^{b}=\left\{{RPE}^{\alpha ,b,n}\right\}$ where α and n indicate respectively video samples and number of ablated units. $N_{s}=10,000$ bootstrap samples are generated by resampling $D^{b}$ with replacement, denoted as $D^{b,s}$ where $s=1,2,\ldots ,N_{s}$. For each bootstrapped dataset, we used ordinary least squares linear regression to compute a slope $k^{b,s}$. 95% confidence interval of the slopes were estimated from the bootstrapped distribution (shown as error bands in Figure 4; Figure S13). Subtracting the two slope sets, we got $N_{s}$ slope differences denoted as $\Delta k^{s}=k^{bos,s}-k^{non-bos,s}$. The *p*-value (two-tailed) was then estimated as $2\times \min\left\{\;Q\left(0,\left\{\Delta k^{s}\right\}\right),1-Q\left(0,\left\{\Delta k^{s}\right\}\right)\;\right\}$ where $Q\left(0,\left\{\Delta k^{s}\right\}\right)$ is the quantile of 0 in the set of slope differences $\{\Delta k^{s}\}$.

### Quantification and statistical analysis

Statistical analyses were performed using Python. Definition of center, dispersion and precision measures are mentioned in figure legends. N and what n represents for population analyses are explained in either the figures or figure legends. We have used a permutation test to assess the statistical significance of *B*_*av*_ (see above “computing selectivity for border ownership”; Figure S2B, S2D, 1D, 1E, S7C, S7D, S7F, S7G, S8C, S8D, S8F, and S8G) and the statistical significance of BOI (see above “variations of square scenes”; Figures 2A and 2B). We have used a Wilcoxon signed-rank test to compare the surround influence between the preferred and the non-preferred side, and to compare the absolute value of surround influence between with-CE scenes and without-CE scenes (Figures 2F and S10). We have used a Wilcoxon signed-rank test to compare the difference in half-life between Square-Ambiguous and Figure-Off sequences (Figure 3D). We have used a bootstrapping test to compare the relative prediction MSE between ablation of BOS units and non-BOS units (see above “computing the prediction error”; Figures 4, S13, and S15). We have used a Wilcoxon rank-sum test to compare cRF sizes between R and E-modules (Figure S6). We have used a bootstrapping test to compare BOI across squares sizes and positions (see above “variations of square scenes”; Figures S9B and S9C). Statistical significance is indicated in the figures as follows: ∗∗∗: *p* < 0.001; ∗∗: *p* < 0.01; ∗: *p* < 0.05.
