# Supplementary material for: Brain-like border ownership signals support prediction of natural videos
Source: iScience. 2025 Mar 11;28(4):112199. doi: 10.1016/j.isci.2025.112199 (PMC11986989; doi:10.1016/j.isci.2025.112199)
Supplement: Document S1. Figures S1–S15 [file mmc1.pdf]

iScience, Volume *28*

Supplemental information

Brain-like border ownership signals support prediction of natural videos

Zeyuan Ye, Ralf Wessel, and Tom P. Franken

Supplemental information

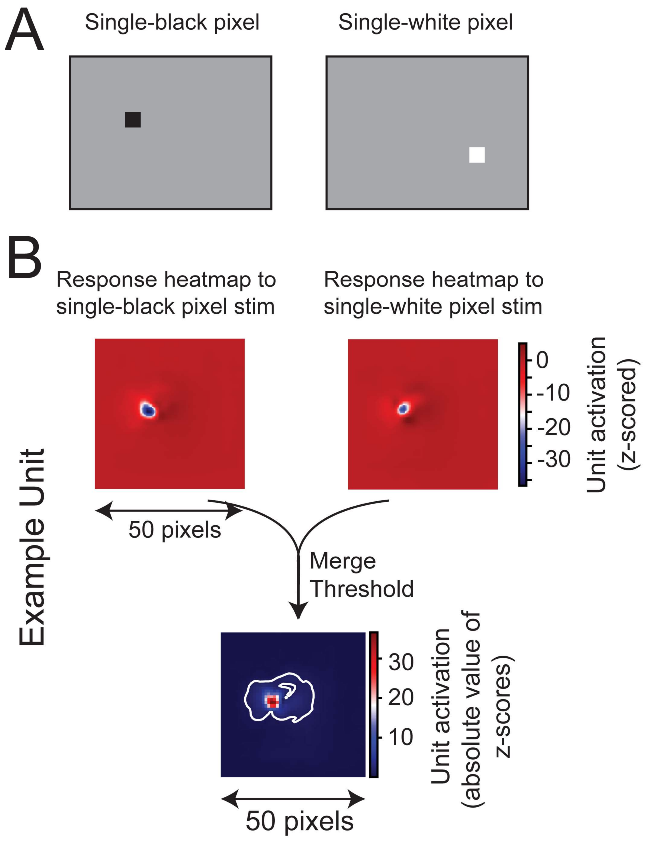

Figure S1. Illustration of the method to measure the cRF of PredNet units, related to Figure 1. (**A**) A sparse noise scene is a gray scene with only one black or white pixel, at a random position. These scenes were used as input to PredNet over four time steps. (**B**) cRF for an example unit. The unit's responses to the sparse noise scenes were collected and normalized (z-scored) into two heat maps, one for black pixel noise and the other for white. Each value in the black or white heatmap corresponds to the unit's normalized response to a black or white pixel at the same entry position. The two heatmaps (for one unit) were merged into one heatmap by taking the maximum absolute values for each entry. Positions with an absolute value of the z-score greater than 1 were defined as the cRF (indicated by white contours).

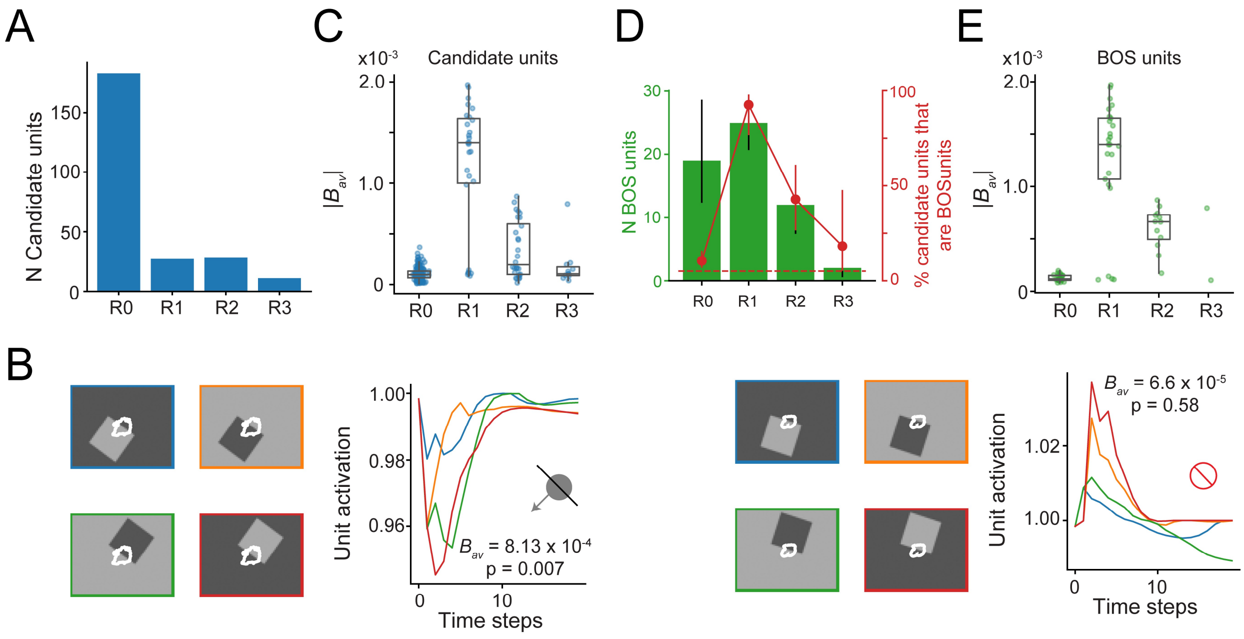

Figure S2. BOS units emerge in PredNet's R modules, related to Figure 1. (**A**) The number of candidate units in R modules across different layers. (**B**) Responses of two example units (module R_2), with white contours indicating the cRF (similar to Fig. 1D). (**C**) The B_{av} distribution of the candidate units in different R modules. Each dot is one candidate unit. Box plot conventions as in Figure 3D. (**D**) Among the candidate units, units with p-value smaller than 0.05 are defined as BOS units. Error bars indicate 95% confidence intervals. Horizontal dashed line indicates chance level of 5%. (**E**) Similar to C, but for BOS units.

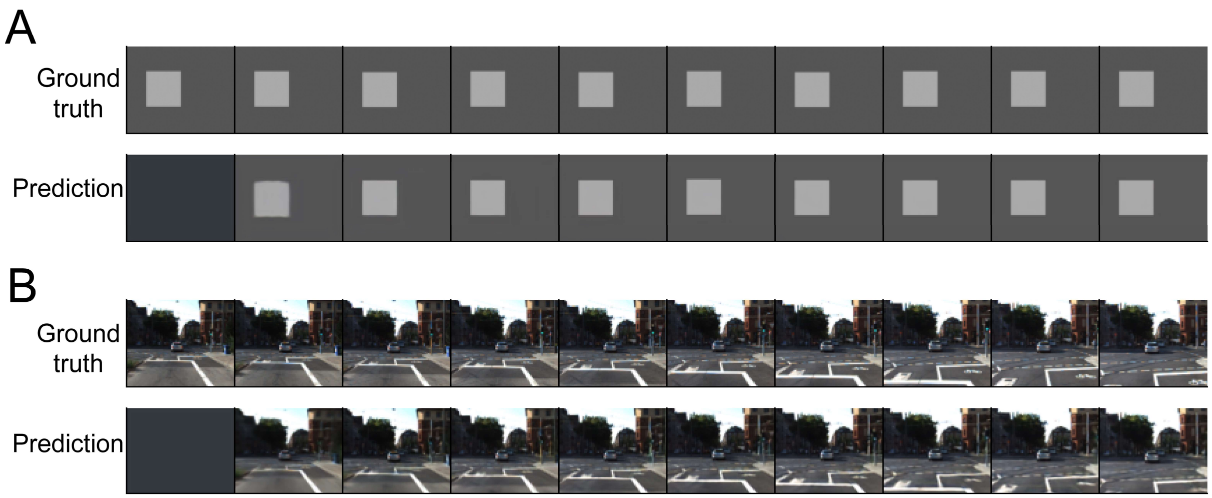

Figure S3. Example stimuli sequences along with PredNet predictions, related to Figure 1. (**A, B**) Static square sequences (e.g., Figure 1D) and test KITTI video (not used for PredNet training). Top row shows ground truth, bottom row shows PredNet predictions. First 10 frames are shown for reasons of space.

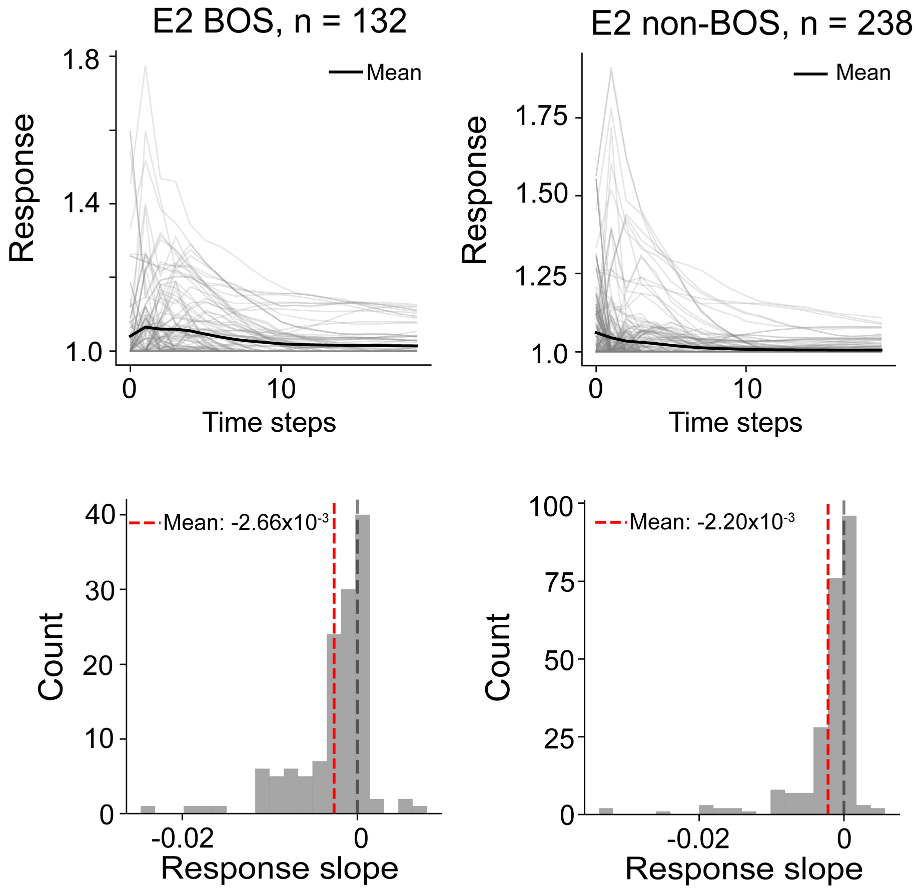

Figure S4. Error module's BOS units' responses to a static square scene, related to Figure 1D. The square (orientation 0°) was repeated for 20 time steps. Upper panels: grey lines are responses of individual units. Panel titles show number of units in the analysis. Lower panels: distribution of response slopes as a function of time (applying ordinary least squares regression over the entire time window) of all BOS or non-BOS units. Black dashed line indicates slope equals zero.

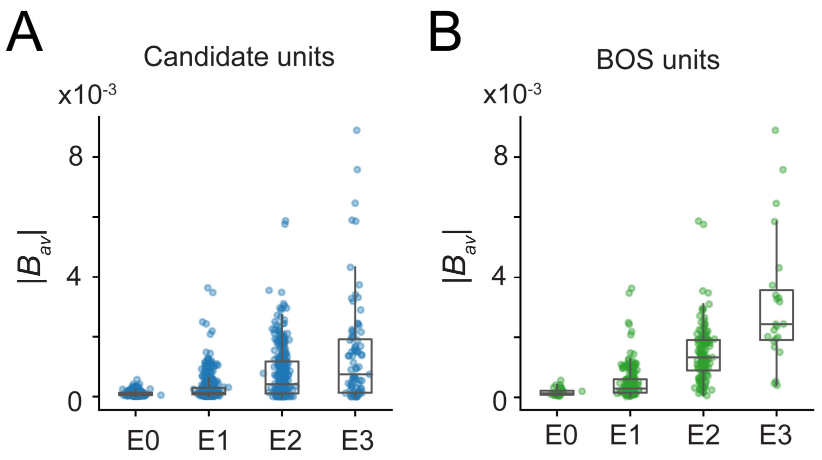

Figure S5. $|B_{av}|$ values of units in E modules, related to Figure 1. Similar to Figure S2C,E, for E modules.

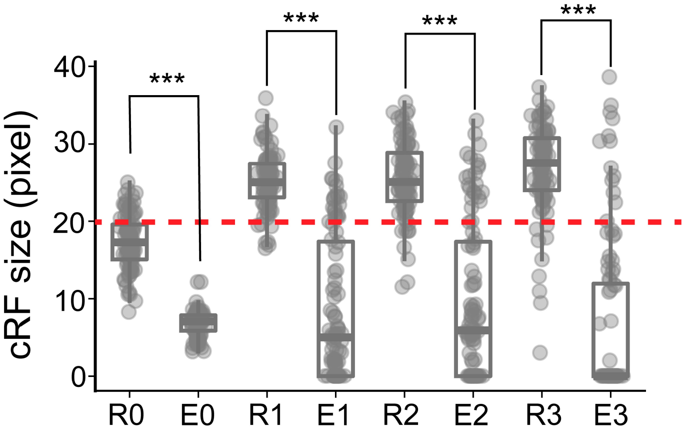

Figure S6. Classical receptive field (cRF) sizes of randomly sampled units across different PredNet modules, related to Figures 1C, 1E. For each PredNet module, we randomly sampled 100 units. The cRF was calculated using sparse noise stimuli (see Methods and Figure S1). The cRF size was defined as the distance from the farthest boundary point of the cRF to its center. Box plot conventions as in Figure 3D. The cRF sizes of units in the R-modules were significantly larger than those in the E-modules within the same layer (***: $p < 0.001$, one-tailed, Wilcoxon rank-sum test). As explained in Methods, cRF size needs to be smaller than 20 pixels (red dashed line) for units to be included in the border ownership analysis.

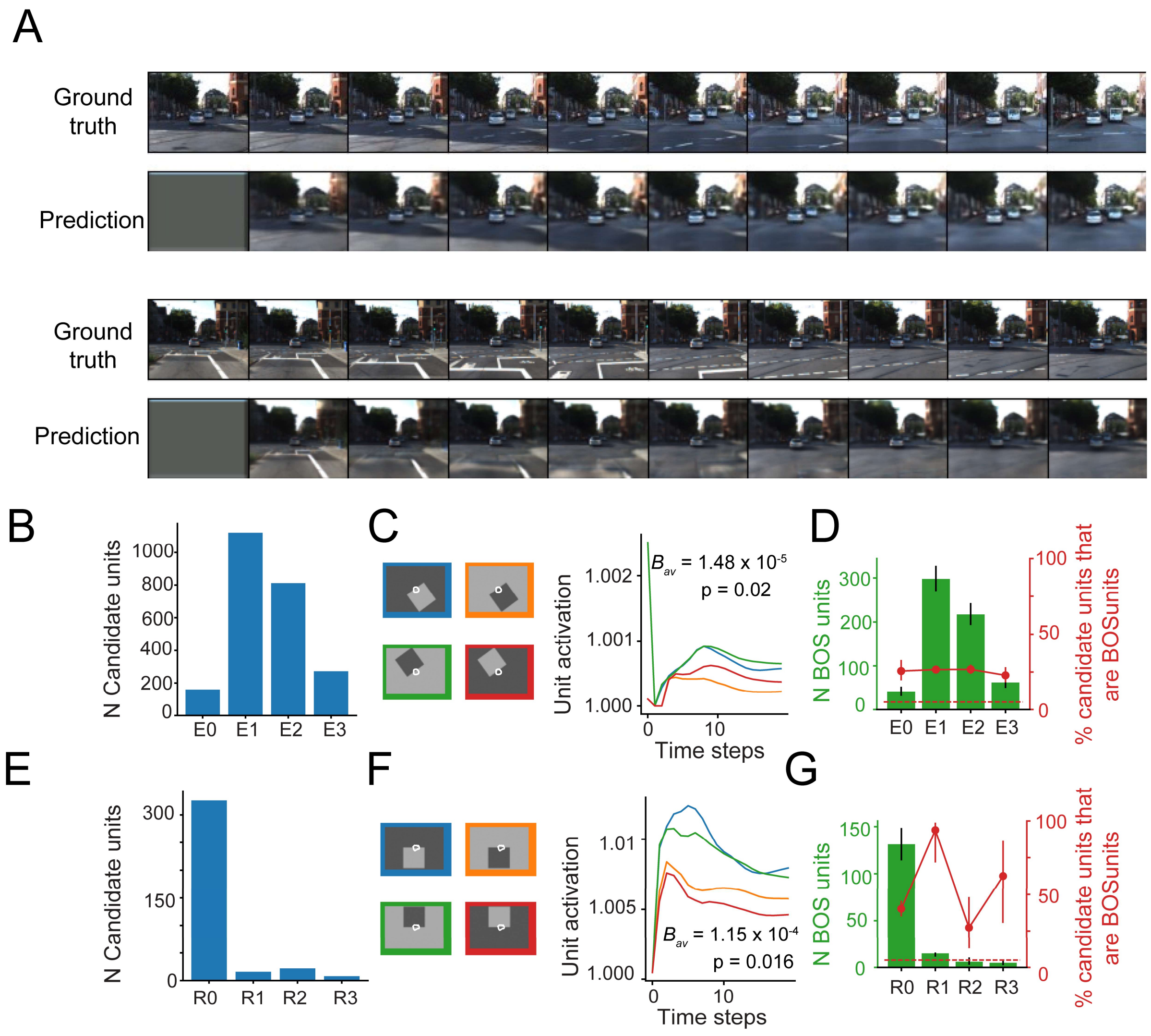

Figure S7. PredNet trained on downsampled videos exhibits BOS signal, related to Figure 1. (**A**) A new PredNet ('DS-PredNet') was trained on downsampled videos (5 frames per second). This panel shows two example predictions. (**B-D**) Similar to Figure 1C-E, for the E modules in DS-PredNet. (**E-G**) Similar to B-D, but for the R modules.

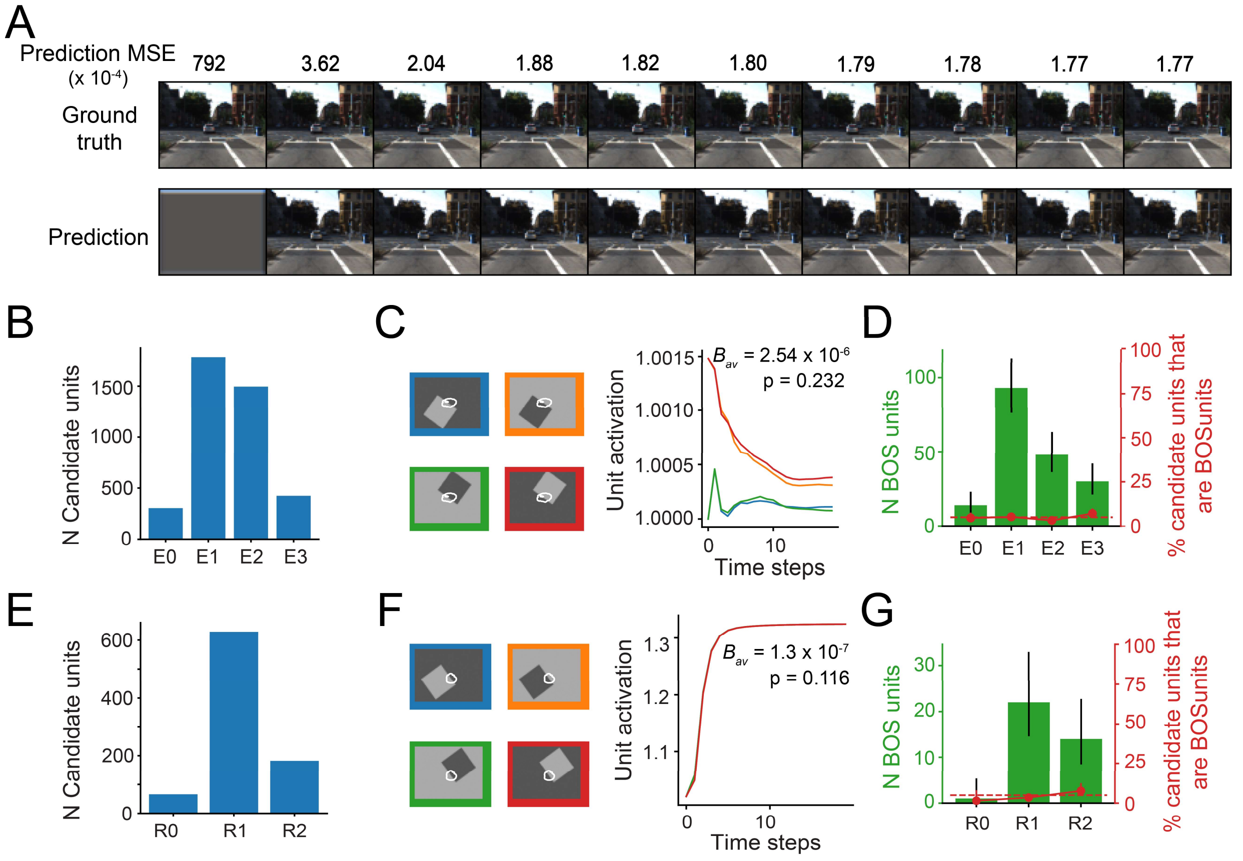

Figure S8. A new instance of PredNet trained to reproduce static images does not have a statistically significant number of BOS units, related to Figure 1. (**A**) Each video frame in the KITTI dataset was repeated 10 time steps and hence becomes a sequence of identical images. We then trained a new instance of PredNet ('Static-PredNet') on this sequence. Since every frame is the same, this is in fact image reproduction. This panel shows an example reproduction of Static-PredNet for one sequence that was not used in training. MSE: mean squared error. (**B-D**) Similar to Figure 1 C-E, for Static-PredNet. C shows a unit from module E2. (**E-G**) Similar to (B-D), but for R modules. F shows a unit from module R2. R3 does not contain candidate units.

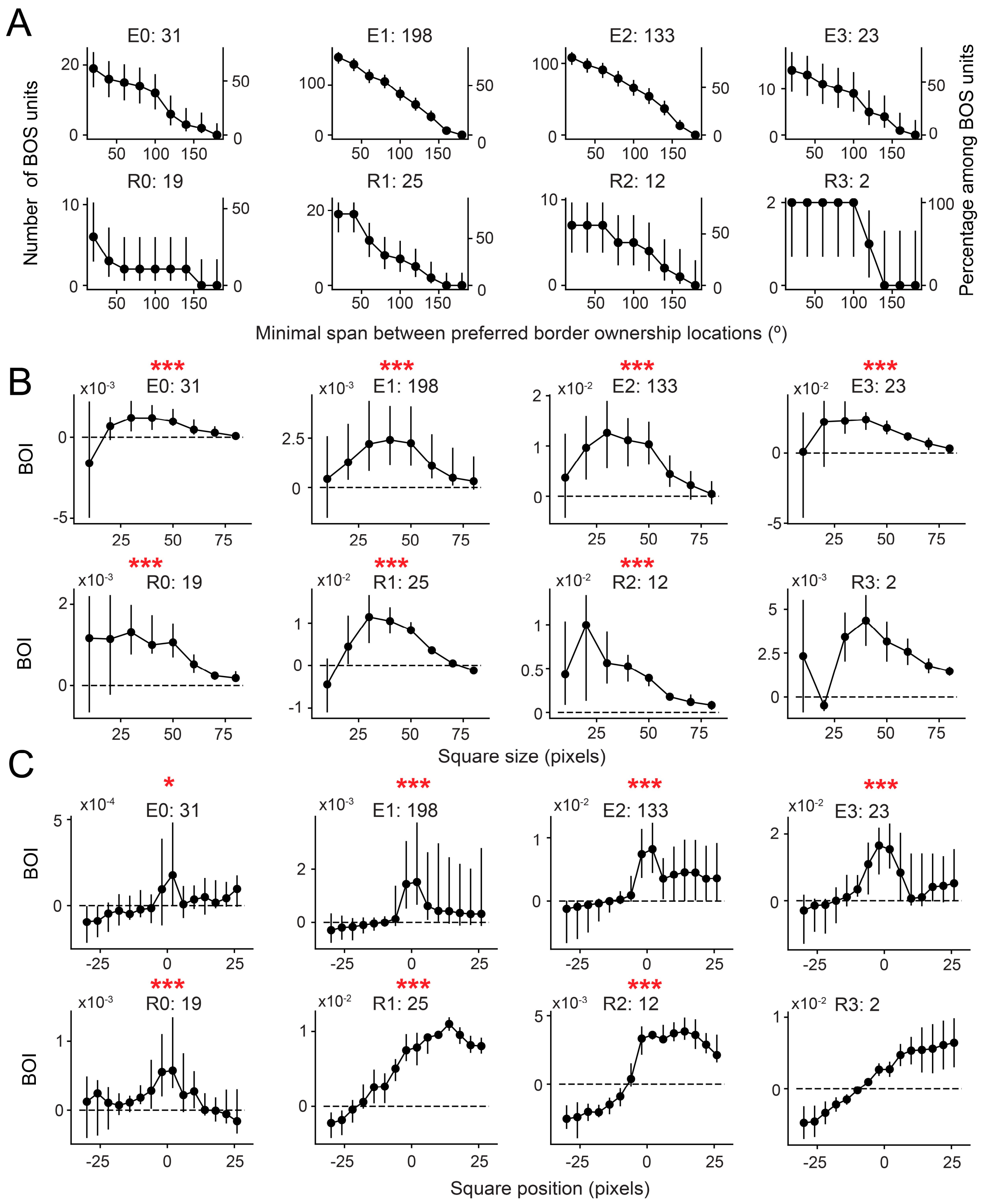

Figure S9. BOS signals are robust to different stimulus parameters, related to Figures 2A-D. (**A**) Similar to Figure 2B, for other modules. (**B, C**) BOI across different square sizes and positions. The dots and error bars represent the median, first and third quartiles across all units in a module. The number after the module name in the panel titles denotes the total number of BOS units included per module. Red symbols indicate whether the averaged BOI across conditions (square sizes or positions) is statistically significantly larger than zero, ***: $p < 0.001$; *: $p < 0.05$; bootstrapping test (see Methods). Statistical significance was only evaluated in modules with more than 10 BOS units.

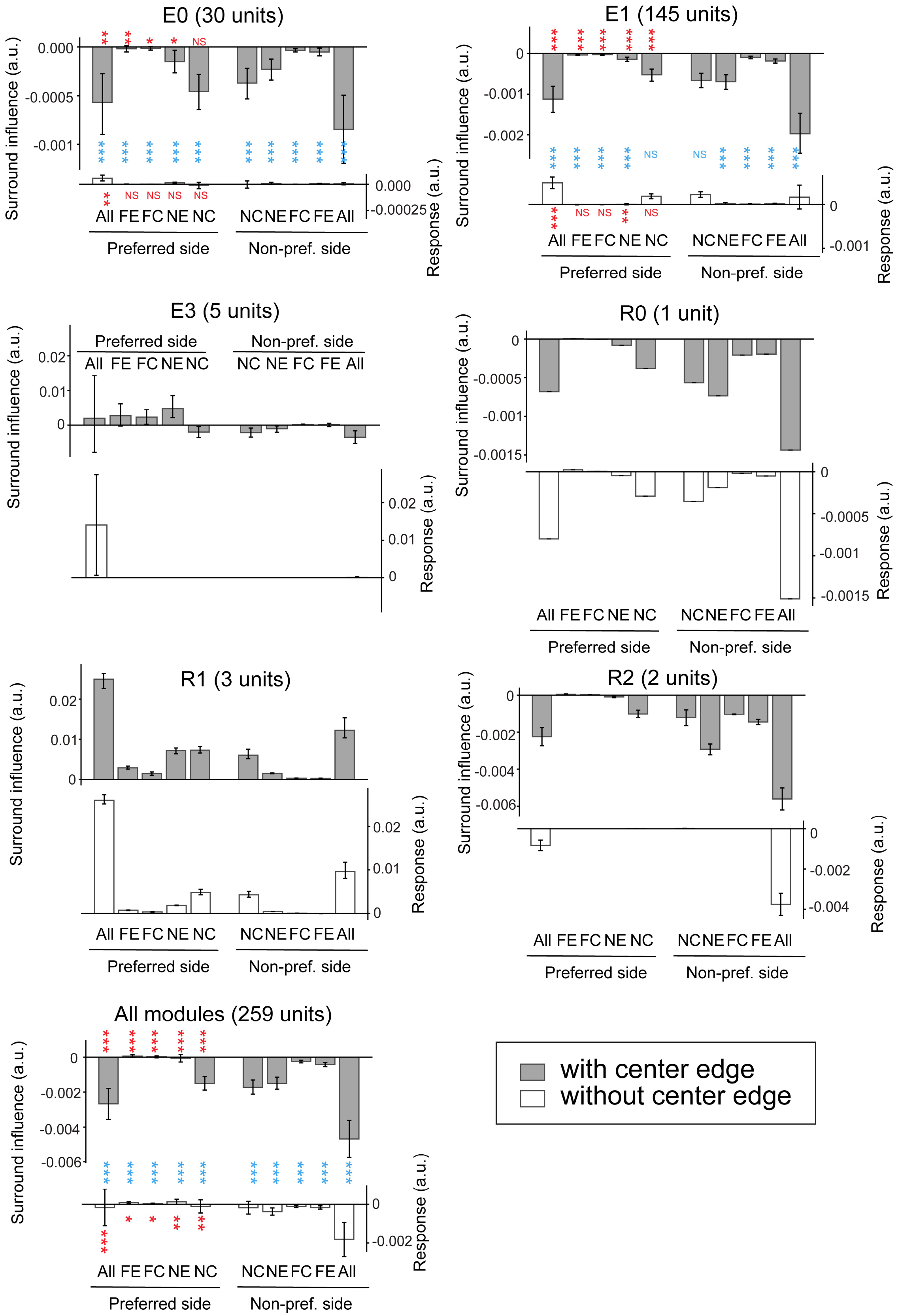

E0 (30 units)
E1 (145 units)
E3 (5 units)
R0 (1 unit)
R1 (3 units)
R2 (2 units)
All modules (259 units)
Surround influence (a.u.)
Response (a.u.)
All FE FC NE NC
NC NE FC FE All
Preferred side
Non-pref. side
NS
0.000
-0.0005
-0.001
-0.00025
0
-0.002
0.02
0.01
-0.0015
-0.004
-0.006
with center edge
without center edge

Figure S10. BOS units' responses to square fragments on the preferred side of BOS are generally larger than on the non-preferred side of BOS, related to Figures 2E-H. Similar to Figure 2F, for BOS units in different PredNet modules. Red text indicates whether the surround influence for a particular condition is significantly larger on the preferred side than on the non-preferred side. To determine whether the magnitude of surround influence in the with-CE condition is greater than in the without-CE condition, we first converted the surround influence of each BOS unit into its absolute value (not shown in this figure), and then conducted a Wilcoxon signed-rank test. ***: $p < 0.001$; **: $p < 0.01$; *: $p < 0.05$; NS: no significance.

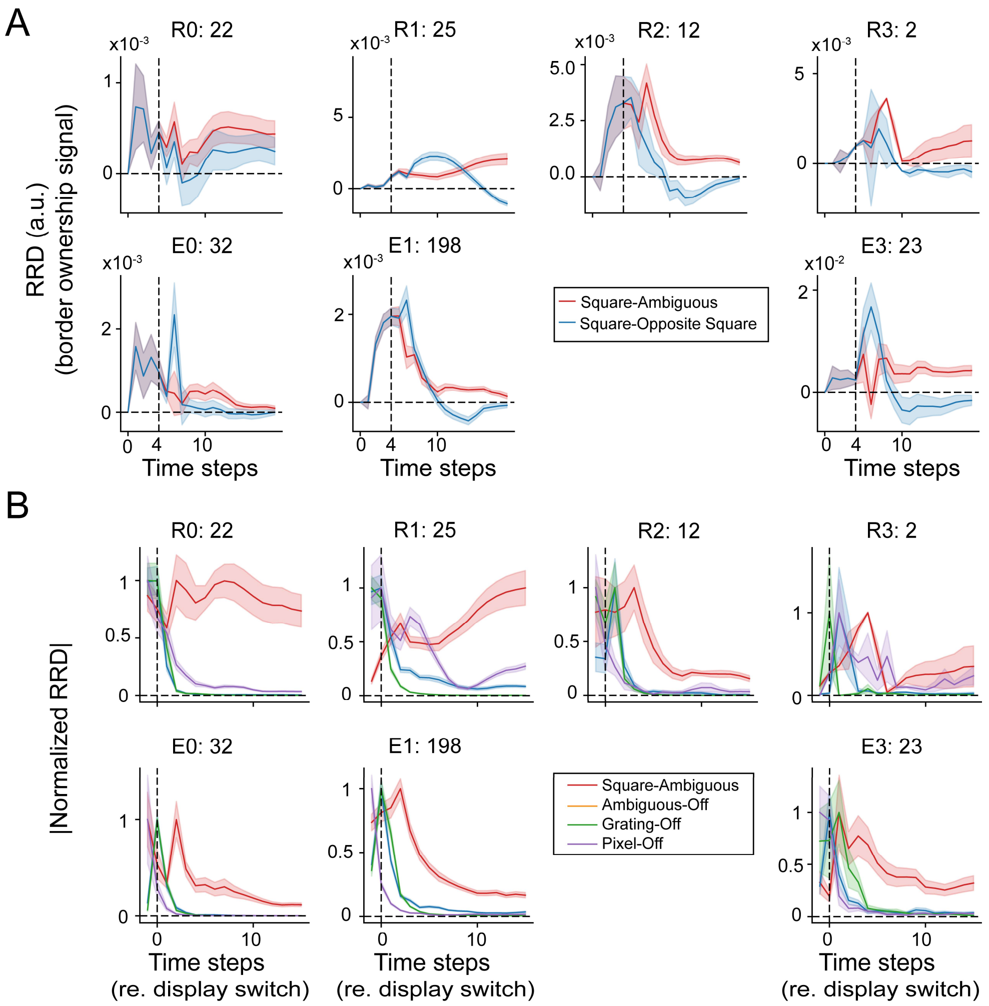

Figure S11. Persistent BOS signals in different modules, related to Figure 3C. (**A**) Similar to Figure 3B, for other modules. The number of BOS units in each module is indicated above each panel. (**B**) Similar to Figure 3C, for other modules.

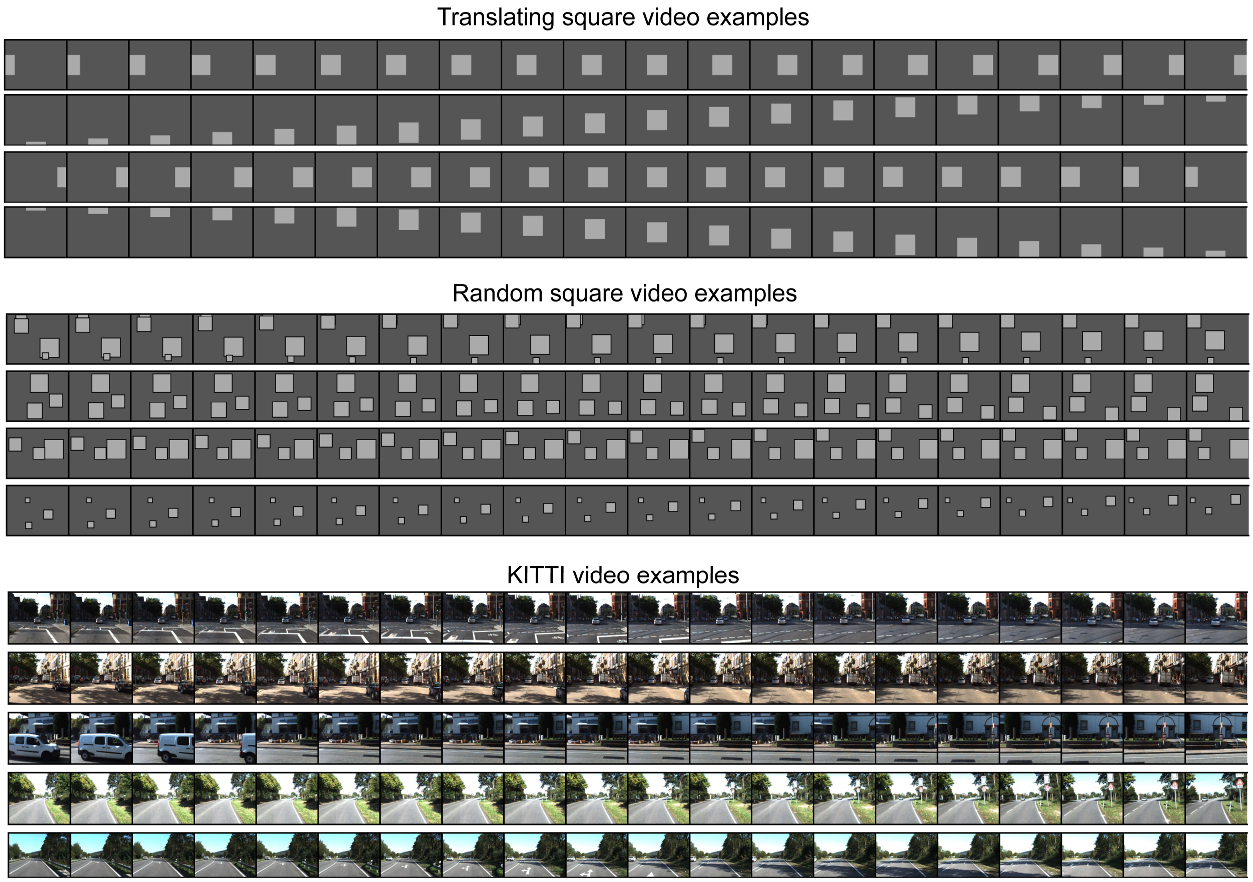

Figure S12. Three video types used for the ablation experiment, related to Figure 4. Figure shows example videos from each video type. Each row shows a different unique video clip for each of the three types. Video length is 20 frames, shown during 20 time steps.

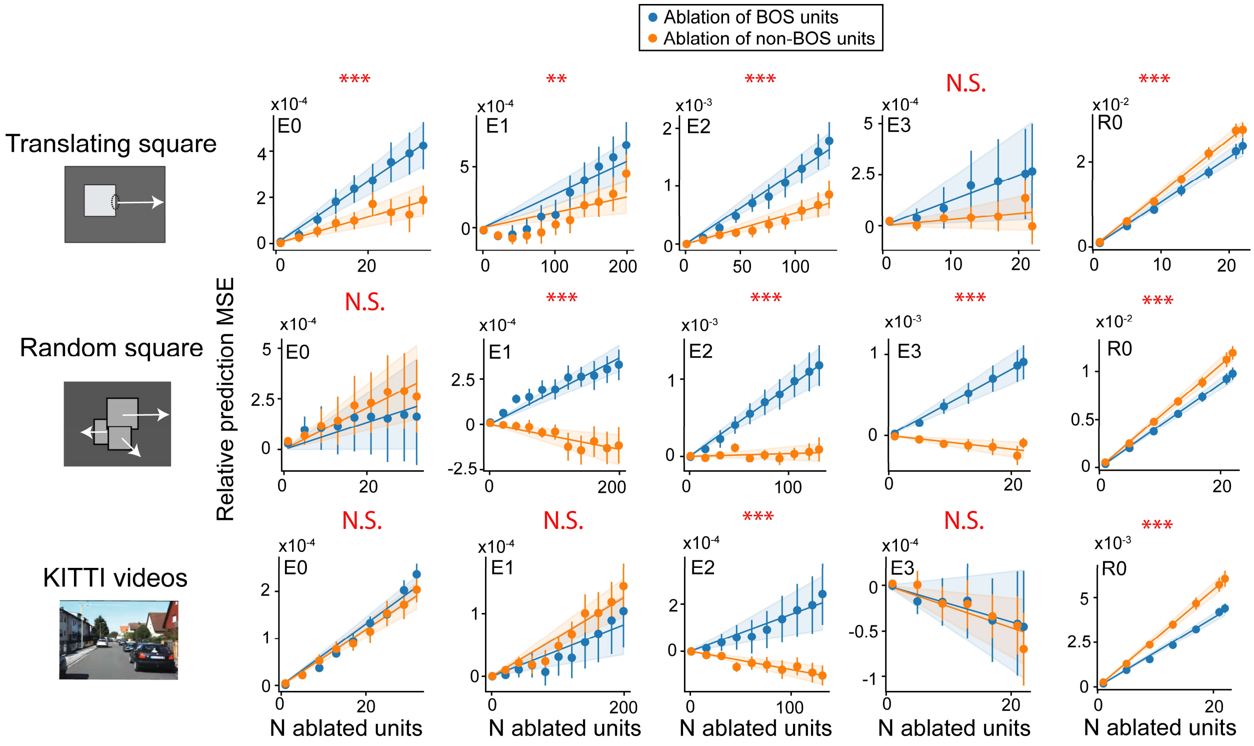

Figure S13. The effect of ablating the original BOS/non-BOS units, without subsampling, related to Figure 4. Similar to Figure 4 but using the original unit population (no subsampling). ***: $p < 0.001$; **: $p < 0.01$; N.S.: not significant (bootstrapping test).

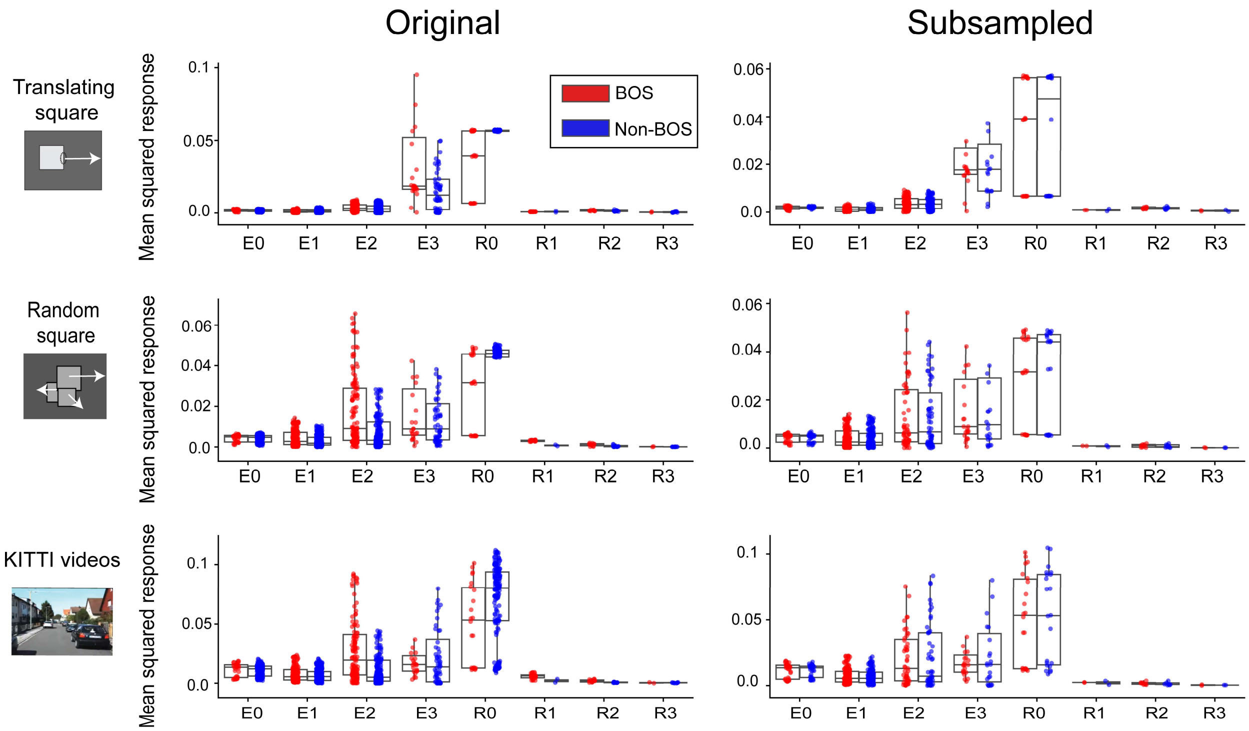

Figure S14. Activity in subsampled BOS and non-BOS unit populations and original populations, related to Figure 4. For each unit, mean squared response is the square of the averaged response, averaged across time and videos. Each dot is one unit's mean squared response. Boxes indicate the interquartile range between the first and third quartiles with central mark inside each box indicating the median. Whiskers extend to the lowest and highest values within 1.5 times the interquartile range from the box boundaries. Outlier units not shown for better visualization (but are included in the metrics indicated by the box plots). The right panels show BOS and non-BOS units after subsampling (see Methods for details on subsampling procedure).

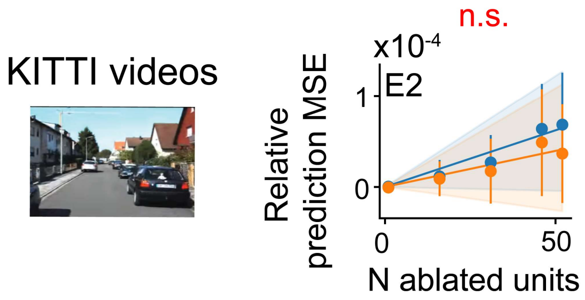

Figure S15. The effect of ablating the subsampled E_2 BOS/non-BOS units on KITTI video prediction, related to Figure 4. Similar to Figure 4, for KITTI videos. n.s.: not significant.
